# PPRC1 is a prognostic biomarker and key regulator of mitochondrial oxidative phosphorylation in multiple myeloma

**DOI:** 10.1080/07853890.2026.2639658

**Published:** 2026-03-11

**Authors:** Yilin Liu, Siqi Yan, Xuemei Shu, Qiang Xu, Min Zhang, Yuxiang Wang, Dawei Wang, Tao Guo

**Affiliations:** a Institute of Hematology, Union Hospital, Tongji Medical College, Huazhong University of Science and Technology, Wuhan, China; b Shanghai Institute of Hematology, State Key Laboratory of Medical Genomics, National Research Center for Translational Medicine at Shanghai, Ruijin Hospital Affiliated to Shanghai Jiao Tong University, Shanghai, China; c Collaborative Innovation Center of Hematology, Huazhong University of Science and Technology, Wuhan, China

**Keywords:** Multiple myeloma, PPRC1, biomarker, oxidative phosphorylation

## Abstract

**Background:**

Multiple myeloma (MM) remains an incurable haematological malignancy, underscoring the need for novel prognostic biomarkers and therapeutic targets. This study aimed to investigate the clinical and biological significance of peroxisome proliferator-activated receptor gamma coactivator-related protein 1 (PPRC1) in MM.

**Methods:**

Expression and clinical data were obtained from public databases and an independent local cohort. Kaplan–Meier and Cox regression analyses were performed to evaluate prognostic value. Differential expression analysis, pathway enrichment analysis and single-cell RNA-seq data analysis were used to explore biological functions. PPRC1 was silenced in MM cell lines using siRNA to assess its effects on cell survival and oxidative phosphorylation.

**Results:**

PPRC1 was significantly upregulated in MM and was associated with advanced disease stage and poor overall survival. Multivariate Cox analysis identified PPRC1 as an independent prognostic factor. A nomogram incorporating PPRC1 and revised-ISS improved survival prediction. Functional analyses revealed that PPRC1 was positively correlated with oxidative phosphorylation and oncogenic signalling pathways. A potential connection between PPRC1 expression and immune cell infiltration was observed. PPRC1 knockdown inhibited cell proliferation, induced cell cycle arrest and apoptosis and impaired oxidative phosphorylation in MM.

**Conclusions:**

PPRC1 acts as a prognostic biomarker and metabolic regulator in MM by sustaining mitochondrial oxidative phosphorylation. These findings highlight PPRC1 as a potential therapeutic target in MM.

## 1. Introduction

Multiple myeloma (MM) is a plasma cell malignancy accounting for approximately 10% of all haematological caners and remains largely incurable despite substantial therapeutic advances. Current frontline treatments include proteasome inhibitors, immunomodulatory drugs, glucocorticoids, alkylating agents, autologous stem cell transplantation and monoclonal antibody-based therapy [1,2]. Although these strategies have significantly prolonged overall survival (OS), most patients eventually relapse and develop drug resistance, underscoring the urgent need to identify novel biomarkers and therapeutic targets.

Energy metabolism reprogramming is a fundamental hallmark of cancer [3]. Beyond the classical Warburg effect, accumulating evidence indicates that oxidative phosphorylation (OXPHOS) plays a critical role in tumorigenesis and tumour progression. OXPHOS has therefore emerged as an attractive metabolic vulnerability in cancer [4–6]. The PGC-1 coactivator family (including PPARGC1A, PPARGC1B and PPRC1) has gained attention as OXPHOS-linked cancer drivers because of its central roles in mitochondrial biogenesis, metabolism and oxidative stress regulation [7,8]. PPARGC1A, one of the most studied genes in this family, has context‑dependent roles in cancer [9,10]. PPARGC1A-driven metabolism regulation has been reported to promote tumorigenesis and progression in melanoma, breast cancer, prostate cancer, ovarian cancer and colorectal cancer [11]. In MM, high expression of mitochondrial biogenesis genes including PPARGC1A correlates with adverse prognosis [12]. PPARGC1A inhibition suppresses tumour growth *via* impairing OXPHOS metabolism [13], while PPARGC1B supports MM growth primarily through glycolysis [14].

However, the biological and clinical relevance of PPRC1 remains largely unexplored. To date, no systematic investigation has evaluated the role of PPRC1 in MM. Therefore, in this study, we conducted a comprehensively analysis of PPRC1 in MM using both multiple public datasets and our local cohort and preliminarily explored its impact on MM cells *in vitro* by cellular and molecular assays. Our findings identify PPRC1 as a novel prognostic biomarker and metabolic regulator in MM.

## 2. Materials and methods

### 2.1. Public datasets and bioinformatic analysis

Transcriptomic and corresponding clinical data of MM were obtained from public databases, including Gene Expression Omnibus (GEO), ArrayExpress and the Multiple Myeloma Research Foundation (MMRF). Differential gene expression analysis, survival analysis, pathway enrichment analysis, immune infiltration analysis, single-cell RNA sequencing analysis and other bioinformatic procedures were performed as described in the Supplementary Methods.

### 2.2. Sample collection

Bone marrow aspirates were collected from 23 newly diagnosed MM (NDMM) patients, 5 relapsed/refractory MM (RRMM) patients, 2 primary plasma cell leukaemia (PCL) patients and 3 anaemia patients with renal dysfunction serving as controls at the Department of Hematology, Union Hospital of Wuhan between 1 January 2024 to 1 May 2025. Peripheral blood mononuclear cell (PBMC) from 7 healthy donors were used as additional controls. After obtaining informed consent, fresh bone marrow (BM) aspirates or peripheral blood (PB) were collected and processed within 2 h. Mononuclear cells were isolated by density-gradient separation (Ficoll-Paque, TBD Science, Tianjin, China) and CD138-positive plasma cells were enriched using CD138 magnetic microbeads (Miltenyi Biotec, CA, USA) according to the manufacturer’s instructions. This study was approved by the Ethics Committees of Union Hospital (No. 2024-1080-01) and conducted in accordance with the current version of the Declaration of Helsinki. Patient clinical characteristics are summarized in Table 1.

**Table 1.** Clinical information of patients in the local cohort.

Disease	NDMM (*n* = 23)	RRMM (*n* = 5)	PCL (*n* = 2)
Characteristics			
Age (<65, ≥65 years)	14, 9	2, 3	1, 1
Gender (female, male)	12, 11	4, 1	1, 1
LDH (<200, ≥200 U/L)	17, 6	4, 1	0, 2
ISS (I, II, III)	4, 7, 12	1, 1, 3	*na*
Gain1q21*^a^* (false, true)	4, 14	3, 2	*na*
High risk CAs*^a^* (false, true)	13, 5	1, 4	*na*

*Notes: na* means no value. *a* contains five missing values. High risk CAs include t (4; 14), t (14; 16) and del17p13.

Abbreviations: NDMM: newly diagnosed multiple myeloma; RRMM: relapsed/refractory multiple myeloma; PCL: plasma cell leukaemia; LDH: lactate dehydrogenase; ISS: International Staging System; CAs: cytogenetic abnormalities.

### 2.3. Cell lines and cell culture

U266, RPMI8226, NCI-H929, MM1S and MM1R were obtained from the Cell Bank of the Chinese Academy of Sciences (Shanghai, China). OPM2 cells were kindly provided by Professor Sun Chunyan (Union Hospital, Hubei, China). All cell lines were authenticated and routinely tested for mycoplasma contamination. Cells were cultured in complete RPMI-1640 medium with 11.1 mM glucose and 2 mM glutamine (Gibco, USA) supplemented with 10% (v/v) foetal bovine serum (ABW, Uruguay) and 1% penicillin/streptomycin (Biosharp, Beijing, China) at 37 °C in a humidified atmosphere with 5% CO2 (Thermo Fisher, California, USA).

### 2.4. Cell viability assay

Cell viability was assessed using a Cell Counting Kit-8 (CCK-8) assay (Topscience, Shanghai, China). Briefly, transfected MM cells (6–10 × 10^3^/well) were seeded into 96-well plates and incubated for 0, 24, 48 and 72 h. At each indicated time point, 10 μl of CCK-8 solution was added to each well, and the cells were incubated for an additional 3 h at 37 °C in the dark. Absorbance at 450 nm was detected using a microplate reader (BioTek, USA). Experiments were performed in triplicate and repeated independently at least three times.

### 2.5. Flow cytometry analysis of cell apoptosis, cell cycle, ROS level and mitochondrial membrane potential

After transfection with siRNA for 48–72 h, cell apoptosis was monitored using the Annexin V- FITC and PI (BD Biosciences, USA) per instructions. The apoptosis rate was defined as the percentage of cells in the Q2 and Q3 quadrants. Cell cycle distribution was assessed using a propidium iodide–based cell cycle assay kit (Elabscience, Hubei, China) according to manufacturer’s instructions. Mitochondrial Membrane Potential (MMP) was detected using JC-1 staining (Beyotime, Shanghai, China) per instructions. The ratio of red to green fluorescence intensity indicated MMP levels. Intracellular reactive oxygen species (ROS) level was measured using a 2′,7′-dichlorofluorescein diacetate (DCFH-DA) fluorescent probe (Beyotime, Shanghai, China) after 28 h of transfection according to the manufacturer’s protocol. The mean fluorescence intensity (MFI) was calculated and normalized to the siNC cells. All samples were analysed using a flow cytometer (BD FACSAria II, USA) and data were processed with FlowJo software (version 10.0, Tree Star, USA).

### 2.6. Intracellular ATP measurement

Intercellular ATP levels were measured using an enhanced ATP assay kit (Beyotime, Shanghai, China) following the luciferin-luciferase method. Briefly, cells were collected and lysed after 48 h of transfection. Subsequently, the supernatant or standard solution was added and mixed with ATP working solution. Total ATP concentrations were calculated based on the luminescence signal standard curve and normalized to the protein concentration, which was determined using the bicinchoninic acid (BCA) assay (Thermo Scientific, USA).

### 2.7. Western blot

MM cells were collected 72 h post-transfection and were lysed in RIPA buffer (Servicebio, Hubei, China) supplemented with PMSF and phosphatase inhibitor (Beyotime, Shanghai, China). Protein samples were separated by SDS-PAGE (Epizyme, Shanghai, China) and transferred to the PVDF membranes (Millipore, USA). After blocking with 5% non-fat milk or bovine serum albumin, membranes were incubated with primary antibodies overnight at 4 °C, followed by incubation with Goat Anti-Mouse (ANT019, AntGene) or Anti-Rabbit (ANT020, AntGene) IgG secondary antibodies. Protein signals were visualized using Enhanced Chemical Luminescence reagent (Biosharp, Beijing, China) with a Gel imaging system (BioRad, USA). ImageJ software was used for the densitometric analysis. Primary antibodies were listed in Table S1.

### 2.8. Quantitative real-time PCR (RT-qPCR)

Total RNA was extracted using RNAiso Plus reagent (Takara, Dalian, China) as per the manufacturer’s protocol. The concentration and purity of extracted RNA were assessed using a NanoDrop 1000 spectrophotometer (Thermo Fisher Scientific, Waltham, MA, USA). Reverse transcription was conducted using the HiScript III RT SuperMix for qPCR (+gDNA wiper) reagent (Vazyme, Jiangsu, China). Quantitative PCR was performed using SYBR Green Master Mix (Vazyme, Jiangsu, China) on an ABI 7500 System (CA, USA). Relative gene expression was calculated using the 2^−ΔΔCT^ method with β-actin as an internal control. The primer sequences are listed in Table S2.

### 2.9. RNA interference

PPRC1 expression was silenced using PPRC1-specific small interfering RNA (siPPRC1). A scrambled siRNA was used as a control (siNC) (OBiO Technology, Shanghai, China). The sequences for the siRNAs were listed in Table S2. OPM2 and RPMI8226 cells were transfected with siRNAs using CALNP^™^ RNAi *in vitro* transfection reagent (D-Nano Therapeutics, Beijing, China) according to the manufacturer’s protocol at final concentrations of 40 and 50 nM, respectively. RT-qPCR and western blot assays were conducted to assess PPRC1 mRNA and protein levels in wild type cells (Blank), transfection reagent control cells (Mock), scramble-transfected cells (siNC) and siPPRC1-transfected cells to evaluate knockdown efficiency at 48 and 72 h post-transfection. The biological effects of PPRC1 knockdown in MM cells were studied within 96 h post-transfection.

### 2.10. Statistical analysis

Kaplan–Meier survival analysis with log-rank test and Cox regression analysis were employed to evaluate the prognostic significance of RRRC1 using the ‘survminer’ and ‘survival’ R packages. The optimal cut point for PPRC1 mRNA in Kaplan–Meier curves was determined using the res. cut function in the ‘survminer’ R package and the visualized maxstat results were provided in Figure S3. Group comparisons were conducted using Student’s *t* test or non-parametric tests as appropriate. The CCK-8 assay was analysed using two-way analysis of variance with Sidak’s multiple comparisons test. Statistical analyses were conducted using R software (version 4.4.0) and GraphPad Prism software (version 8.0). A two-sided *p* value < .05 was considered statistically significant.

## 3. Results

### 3.1. PPRC1 is upregulated in MM and is associated with poor prognosis

We initiated our study by exploring PGC-1 family members, including PPARGC1A, PPARGC1B and PPRC1, across normal and pan-cancer tissues. As shown in Figure S1A and 1B, the expression of all PGC-1s genes varies across different tissues, while PPRC1 displays the highest expression in most normal and cancerous tissues. Besides, PPRC1 exhibited elevated expression in the majority of primary, recurrent and metastatic tumour tissues relative to their normal controls (Figure S1C), and a negative correlation was observed between PPRC1 expression and OS in pan-cancer patients (Figure S1D). Notably, while PGC-1s expression in normal CD138-positive enriched bone marrow cells is relatively low among normal tissues, its level in MM patients ranks among the highest across pan-cancer tissues. These findings implicate PPRC1 in cancer development, potentially extending to MM.

**Figure 1.**
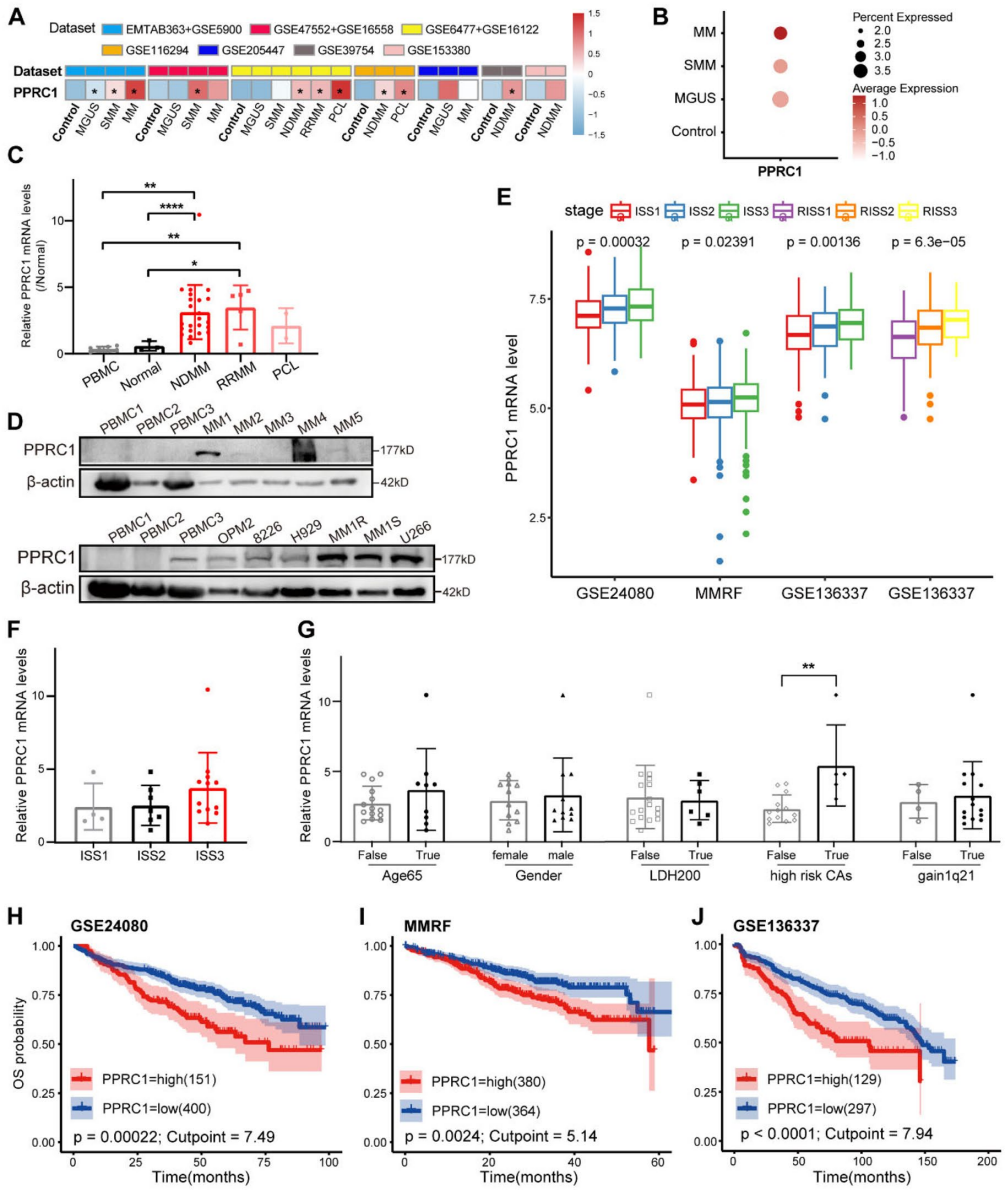
PPRC1 is upregulated in MM and correlated with poor prognosis. (A) Heatmap showing the normalized mean mRNA level of PPRC1 for healthy controls and plasma cell disease patients in 10 public datasets from GEO and ArrayExpress databases. (B) Dotplot showing the expression level and percentage of PPRC1 in plasma cells across different disease status in GSE193531 dataset. (C) Comparison of PPRC1 mRNA levels in PBMC from seven healthy donors, normal plasma cells from three renal anaemia donors, CD138+ tumour cells from 23 NDMM, five RRMM and two PCL patients from our centre detected by RT-qPCR. (D) The protein levels of PPRC1 in PBMC from healthy donors, malignant plasma cells from MM patients and HMCLs were detected by WB. β-actin is included as a loading control. Comparison of PPRC1 mRNA level in distinct MM disease stages of NDMM patients in public datasets (E) and our local cohort (F). (G) Comparison of PPRC1 expression levels in NDMM patients with distinct age (age ≥ 65 years), gender, LDH (LDH ≥ 200 U/L), high risk CAs (t (4; 14), t (14; 16), del17p13) and gain1q21 status from our cohort. PPRC1 is significantly higher in NDMM patients with high risk CAs. (H-J) Kaplan–Meier survival curves of overall survival in NDMM patients based on a high or low PPRC1 mRNA level stratified by the optimal cutpoint using ‘surviminer’ R package. PBMC, peripheral blood mononuclear cell; MGUS, monoclonal gammopathy of undetermined significance; SMM, smoldering myeloma; MM, myeloma; NDMM, newly diagnosed MM; RRMM, relapsed/refractory MM; PCL, plasma cell leukaemia; CAs, cytogenetics abnormalities. *p < .05 compare to control.

We then collected available MM transcriptomic datasets from GEO and ArrayExpress to systematically analyse PPRC1 expression in plasma cell diseases. PPRC1 expression was significantly higher in NDMM and RRMM than in normal controls (*p* < .05). Increased expression was also observed in monoclonal gammopathy of undetermined significance (MGUS), smoldering MM (SMM) and PCL (Figure 1(A,B), Figure S2A). We further confirmed that PPRC1 is significantly elevated in CD138-positive malignant plasma cells from NDMM patients and RRMM patients compared to normal plasma cells and PBMC from healthy donors (*p* < .05) (Figure 1(C)) at our local centre. Although patients with PCL showed similar trends, statistical significance was not achieved because of the small sample size. WB analysis also revealed higher PPRC1 protein levels in MM patients and human myeloma cell lines (HMCLs) than in normal PBMC (Figure 1(D)). Collectively, these results indicate that PPRC1 overexpression is closely associated with MM.

We subsequently analysed the relationships between PPRC1 and clinicopathological characteristics of MM patients. PPRC1 were significantly higher in patients with advanced International Staging System (ISS) or revised ISS (RISS) stages (Figure 1(E)), cytogenetic abnormalities (CAs), high-risk molecular subgroups and relapsed disease (Figure S2B–D) than in their respective controls (*p* < .05). The tumour mutation burden (TMB), an established risk factor for cancers including MM (Figure S2E**–**F), also showed a significant positive correlation with PPRC1 expression (Figure S2G) and TP53, FAM46C and TRRAP mutations were more frequent in the high-PPRC1 group (Figure S2H). We observed similar clinical correlations in our local cohort, namely, an increasing expression trend in patients with ISS3, advanced age, high-risk CAs and gain of 1q21, with high-risk CAs [15] showing a statistically significant difference (*p* < .01) (Figure 1(F–G)). In summary, our findings suggest a positive correlation between PPRC1 expression and aggressive clinical features in MM.

To evaluated the prognostic value of PPRC1 in MM, we divided MM patients into high and low expression groups on the basis of the optimal cut-off point. Kaplan–Meier analysis revealed that high PPRC1 expression was linked to poor OS in MM patients (*p* < .01) (Figure 1(H–J)) in three independent NDMM datasets. Similar results were also observed in two RRMM datasets (*p* < .01) (Figure S2I–J). Moreover, univariate Cox regression analysis identified PPRC1 as a significant risk factor (HR > 1, *p* < .05) for OS and progression-related outcomes across multiple tested datasets (Table S3). Overall, PPRC1 expression serves as an adverse prognostic factor for patients with MM.

### 3.2. PPRC1 is an independent risk factor for MM

**Figure 2.**
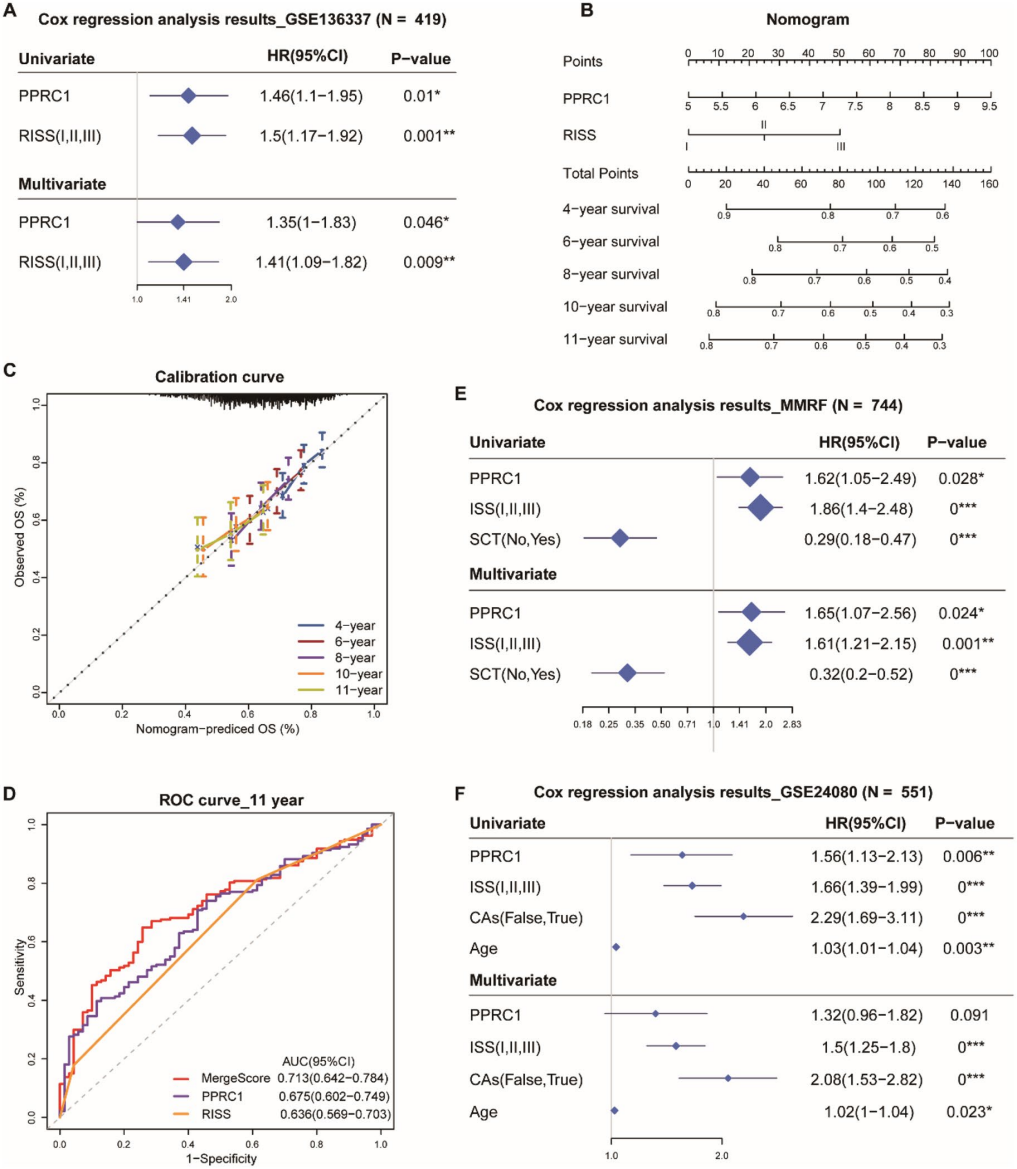
PPRC1 is an independent and promising prognostic factor in MM. (A) The forest plot shows the results of univariate and multivariate Cox regression analyses of PPRC1 expression and revised ISS (RISS) by OS in GSE136337 dataset using ‘forestplot’ R package. (B) The nomogram model incorporating PPRC1 and RISS to predict the OS of MM patients from 4 to 11 years by the ‘rms’ R package. (C) The calibration curves for the nomogram model. The observed lines were closed to the diagonal dashed line at all indicated timepoints. (D) The ROC curves showing the 11-years overall survival AUC of PPRC1, RISS and their combined model (MergeScore). The forest plots showing the results of Cox regression analyses of PPRC1 expression and classic prognostic factors by disease-free survival in MMRF (E) and OS in GSE24080 (F) datasets.

To further investigate the predictive independence of PPRC1 in MM, univariate and multivariate Cox regression analyses were conducted. The widely accepted RISS system, based on cytogenetic abnormalities and serum biomarkers, served as the clinical benchmark. As illustrated in Figure 2(A), both PPRC1 and RISS emerged as significant risk factors according to univariate and multivariate analyses (HR > 1, *p* < .05) in the GSE136337 dataset, solidifying PPRC1’s status as an independent risk factor for OS in MM patients. Given the considerable heterogeneity in MM patients, we established a nomogram model incorporating PPRC1 and RISS to estimate the OS in MM. Points for PPRC1 and RISS were combined to derive total points, predicting OS rates from 4 to 11 years (Figure 2(B)). Calibration curves demonstrated good agreement between predicted and actual observed survival probabilities, highlighting the model’s predictive power (Figure 2(C)). Additionally, we evaluated the predictive accuracy of PPRC1 and RISS alone or in combination using time-dependent ROC curve and AUC analyses. The combined model integrating PPRC1 with RISS (MergeScore) yielded a superior AUC compared to either variable alone (Figure 2(D)), demonstrating that their integration enhances the efficiency of OS prediction. Similarly, PPRC1 retained significant prognostic value in multivariate Cox analysis in the MMRF dataset and approached significance in the GSE24080 dataset (Figure 2(E,F)). These results confirmed that PPRC1 has independent predictive value for prognosis in MM patients.

### 3.3. Identification of PPRC1-related genes and functions in MM

**Figure 3.**
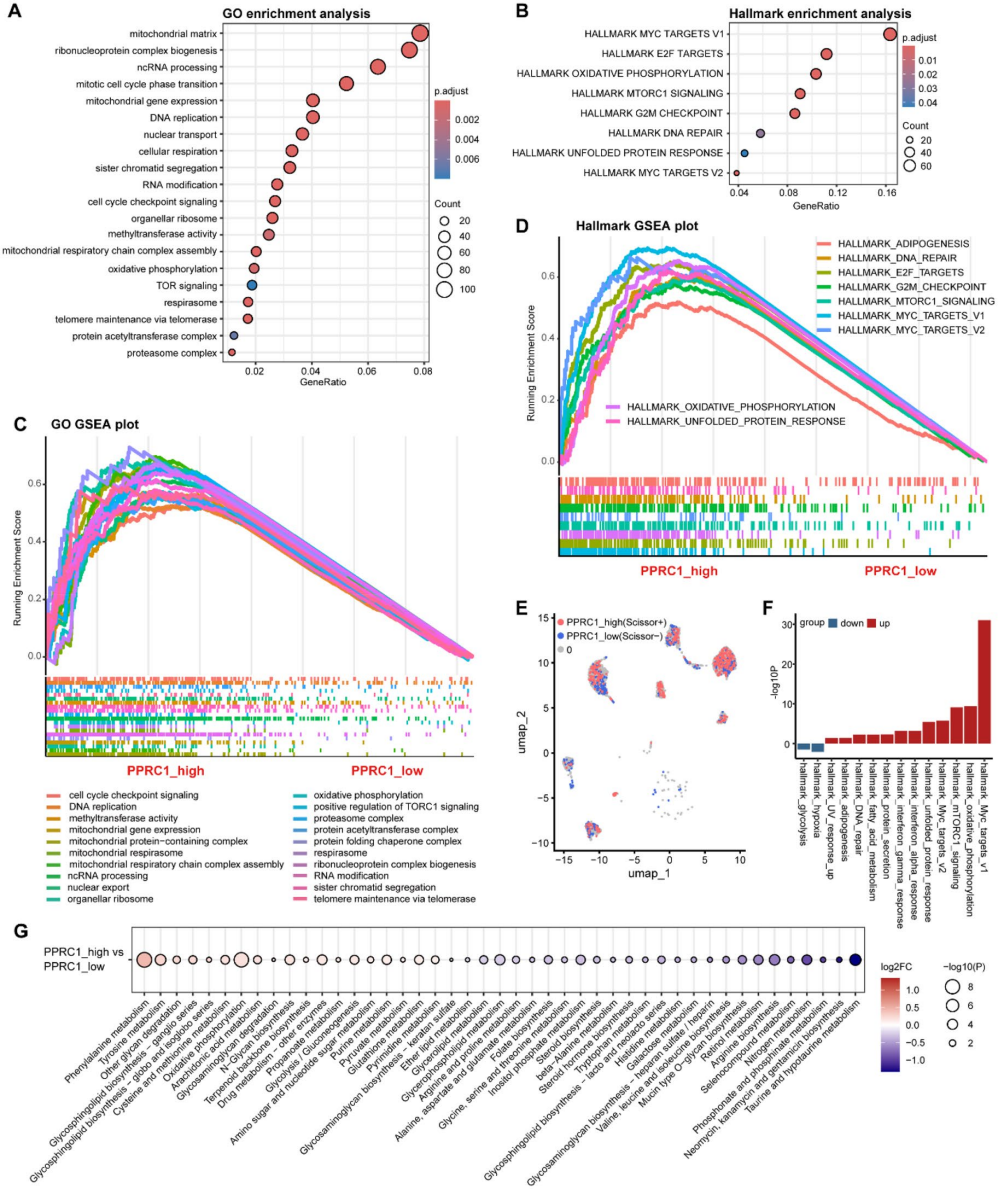
PPRC1-related genes primarily involve in gene expression and OXPHOS metabolism pathways in MM. Bubble plot of Gene Ontology (GO) (A) and Hallmark (B) functional enrichment analysis of PPRC1-related DEGs (fold change >1.5, adjust *p* value < .05) in PPRC1 high versus low expression groups in GSE136337. (C) GSEA plot exhibited interested top GO terms significantly enriched in PPRC1 high expression group. The gene number range was set to 10 to 500. (D) GSEA plot exhibited Hallmark terms significantly enriched in PPRC1 high expression group. (E) UMAP visualization of PPRC1_high (Scissor+) (*n* = 421) and PPRC1_low (Scissor-) (*n* = 392) MM cells in GSE193531 scRNA-sequencing dataset by ‘Scissor’ algorithm. PPRC1 expression subgroup in GSE136337 dataset was set as interest phenotype. (F) Barplot showing Hallmark functional enrichment analysis of significant different markers in PPRC1_high vs PPRC1_low cells in GSE193531 dataset. The red and blue colour indicated the enrichment results of significantly upregulated and downregulated genes, respectively. (G) Bubble chart illustrating the comparison result of metabolism score between PPRC1_high and PPRC1_low cells identified in GSE193531 dataset by scMetabolism using KEGG pathway.

To investigate the biological function associated with PPRC1, we performed differentially expressed gene (DEG) analysis to identify PPRC1-related genes. MM patients were stratified into tertiles (low, median and high expression) based on PPRC1 levels. A total of 1513 DEGs were identified between high and low expression groups in the GSE136337 dataset. GO enrichment analysis showed significant enrichment in genetic information processing, metabolism and cell cycle-related pathways (Figure 3(A)). GSEA based on GO terms identified OXPHOS and gene expression as pathways positively correlated with PPRC1 expression (Figure 3(C)), suggesting OXPHOS as a likely downstream regulatory axis. Hallmark functional enrichment and GSEA revealed oncogenic and proliferative pathways, including ‘MYC TARGETS’, ‘E2F TARGETS’, ‘MTORC1 SIGNALING’ and ‘G2M CHECKPOINT’, were enriched in the high PPRC1 expression group, reinforcing its tumour-promoting role in MM (Figure 3(B,D)). Similar results were obtained in the GSE24080 dataset (Figure S4A and 4C). Moreover, the scissor algorithm was used to identify the most relevant malignant cell populations contributing to the observed differences between the PPRC1 expression subgroups. In our investigation, PPRC1_high patients and PPRC1_low patients were designated as our primary phenotypes and the corresponding transcriptomic data within the GSE136337 bulk RNA-sequencing dataset was used to link each cell in the GSE193531 single-cell RNA-sequencing dataset. A total of 421 PPRC1_high cells (Scissor + cells) and 392 PPRC1_low cells (Scissor- cells) were identified (Figure 3(E)). The reliability test revealed a *p* value less than .05, indicating a reliable phenotype-cell association. Hallmark functional analysis of the DEGs between the PPRC1_high and PPRC1_low cells revealed that metabolism-related pathways including OXPHOS were significantly enriched in the PPRC1_high cells (Figure 3(F)). The metabolic activity of most pathways related to scMetabolism differed distinctly between the PPRC1-associated Scissor subpopulations, confirming the pivotal role of PPRC1 in MM metabolism (Figure 3(G)).

In conclusion, these findings suggest that PPRC1 may exert onco-promoting effects by influencing OXPHOS.

### 3.4. Tumour immune infiltration by PPRC1 in MM

**Figure 4.**
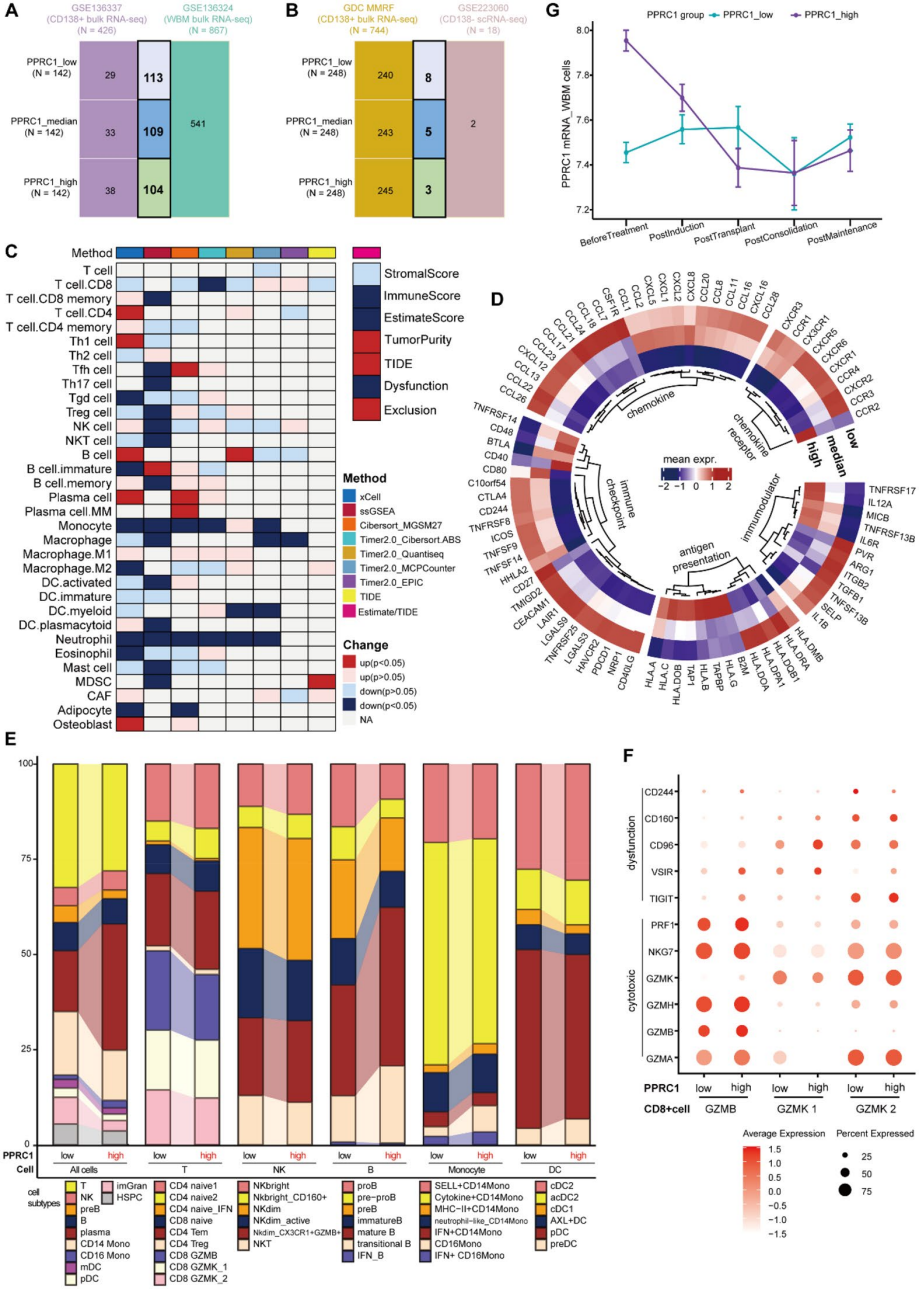
Immune infiltration analysis of PPRC1 in MM. A schematic representation showing the grouping method and sample information used in GSE136324 (A) and GSE223060_MMRF cohort (B). Patients were grouping based the on the left CD138+ tumour cell samples. Patients with median PPRC1 expression were categorized into PPRC1 high group in GSE223060_MMRF cohort due to small sample size. (C) Comparison of infiltrating immune cells and immune score estimated by various deconvolution algorithms between PPRC1 expression subgroups in GSE136324. Statistical differences were assessed with nonparametric tests. Colour-coded are as follows: red (increased in PPRC1 high group), blue (decreased) and grey (not applicable). (D) The circle heatmap showing the mean expression of immune-related molecules with significant difference between PPRC1 subgroups in GSE136324. (E) Stacked boxplot showing proportions of infiltrating immune cell subtypes for PPRC1 low and high group in GSE223060_MMRF scRNA-sequencing cohort. (F) Dotplot showing gene expression of cytotoxic and dysfunction related makers in cytotoxic CD8+ T cells for PPRC1 low and high expression group. (G) PPRC1 expression changes in the longitudinal WBM samples across different timepoints in the high and low PPRC1 groups. TIDE: tumour immune dysfunction and exclusion; preB: precursor B cell; Mono: monocyte; mDC: myeloid dendritic cell; pDC: plasmacytoid dendritic cell; imGran: immature granulocyte; HSPC: haematopoietic stem and progenitor cell; Tem: effector memory T cell; Treg: regulatory T cell; proB: progenitor B cell; cDC: classical DC.

Building on the enrichment of immune pathways in PPRC1-related genes in the MMRF dataset (Figure S4B and 4D), we further explored the relationship between PPRC1 and immune cell infiltration in MM using both bulk and single cell RNA-seq data. As shown in Figure 4(A,B), we stratified patients into tertiles based on PPRC1 levels in CD138^+^ tumour cells and analysed matched whole bone marrow (WBM) samples with multiple deconvolution algorithms. Patients with high PPRC1 expression exhibited a more compromised TME, characterized by higher TIDE and exclusion scores, lower immune and ESTIMATE scores and reduced infiltration of key immune cells (e.g. CD8^+^ memory T cells, NK cells, activated dendritic cells, monocyte, macrophage, neutrophil) (Figure 4(C)). In addition, our comparative analysis revealed lower expression of most of the analysed immune-related genes in high-PPRC1 patients (Figure 4(D)).

Recognizing the constraints of deconvolution algorithms, evidenced by inconsistent outputs in Figure 4(C), we adopted scRNA-seq analysis of CD138-negative bone marrow mononuclear cells (BMMNC) from the GSE223060_MMRF cohort and ultimately identified 11 major cell types (Figure S5A–B). In line with deconvolution analysis of bulk RNA-seq data, the results of the scRNA-seq data revealed an elevated proportion of plasma cells and a diminished proportion of granulocytes in patients with high-PPRC1 patients (Figure 4(E)). The proportion of T cell, precursor B cell and haematopoietic stem and progenitor cell (HSPC) were lower in PPRC1 high group. Subclustering analysis further suggested an immunosuppressive TME characterized by a higher proportion of the naive CD4 T cell, NKbright cell, CD16 monocyte and precursor DC, lower cytotoxic CD8 T cell (GZMB+/GZMK+ CD8 cell) and other immune subsets (e.g. active NKdim cell, NKT cell, cytokine + CD14 monocyte, cDC1 and plasmacytoid DC) (Figure 4(E)). In addition, cytotoxic CD8 T cell in high-PPRC1 patients expressed higher level of markers of exhaustion (TIGIT, VSIR, CD244), indicating an accumulation of dysfunction T cells (Figure 4(F)). In conclusion, these findings illustrated that PPRC1 is connected to TME in MM.

Finally, we explored the expression profile of PPRC1 in infiltrating immune cells in MM. PPRC1 expression was elevated across most immune cell types in MM patients compared to healthy donors (Figure S5C), while patients in the high PPRC1 group showed markedly reduced PPRC1 mRNA level in WBM samples after treatment (Figure 4(G)). Taken together, these findings suggest that PPRC1 might be a potential indicator of tumour burden and a participant in immune reprogramming in MM.

### 3.5. Gene variation and regulatory network analysis of PPRC1 in MM

**Figure 5.**
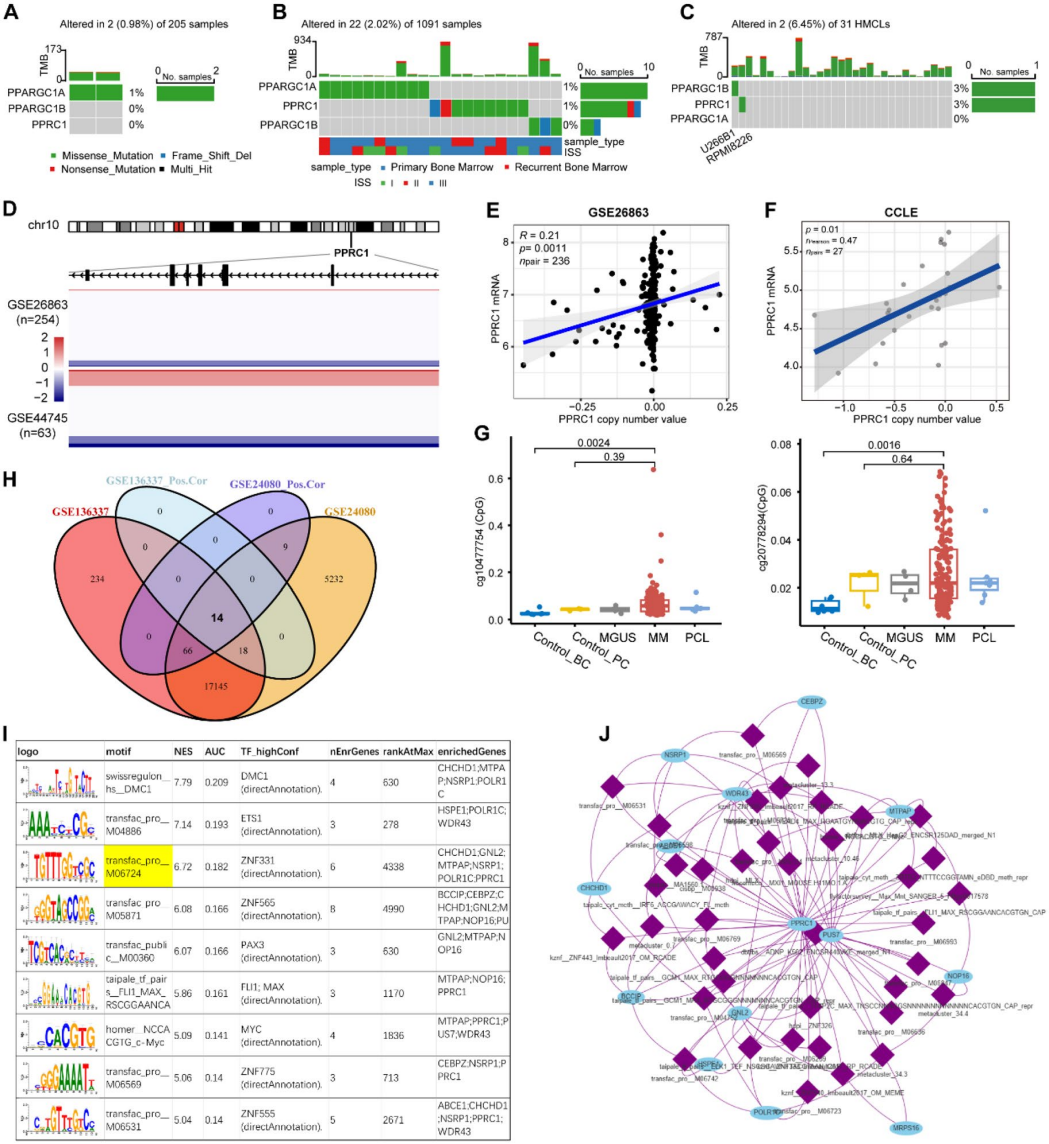
Gene variation and regulatory network analysis of PPRC1 in MM. The oncoplots showing PGC-1s gene mutation information in MMRC dataset from cBioportal (A), MMRF dataset from GDC (B) and HMCLs from CCLE (C). (D) Heatmaps of copy number profiles in PPRC1 genomic region in GSE26863 and GSE44745 datasets. Colour intensity indicates the corresponding degree of copy number gains (red), diploid state (white) or losses (blue). Scatter plot showing PPRC1 copy number and mRNA level in MM patients from GSE26863 (E) and HMCLs from CCLE (F). (G) Comparison of DNA methylation level of two PPRC1 gene loci in normal and malignant plasma cells in GSE21304. (H)Venn diagrams representing the number of PPRC1 strong positive co-expressed genes in GSE136337 and GSE24080 datasets. Pearson coefficient R^2^ > 0.6 and *p* < .05 were used as significant thresholds. (I) Data table showing the top 5 scoring motifs and top 5 motifs enriched for genes including PPRC1. PPRC1 strong positive co-expressed genes were used as key genes for motif enrichment analysis by ‘RcisTarget’ R package. (J) Network plot showing the connection between 39 motifs for which PPRC1 is a predicted target gene and their enriched genes.

We investigated the potential mechanisms underlying the dysregulation of PPRC1 expression in MM by examining gene variations and methylation. Genomic analysis revealed that the mutation frequency of PPRC1 was rare in MM patients and cell lines (Figure 5(A–C)). Although copy number gain was rare (Figure 5(D)), a positive correlation between PPRC1 expression and copy number was detected in MM patients (R2 = 0.21, *p* = .0011) (Figure 5(E)) and cell lines (R2 = 0.47, *p* = .01) (Figure 5(F)). In parallel, DNA methylation levels at the PPRC1 locus did not differ significantly between normal and MM plasma cells (Figure 5(G)). Interestingly, PPRC1 was identified with increased mRNA levels and hypomethylated promoter regions in MM patients with t(11;14) relative to normal controls [16]. However, this reported hypomethylation did not correspond to detectable differences at individual CpG sites. In summary, gene variation and methylation may not be responsible for the high expression of PPRC1 in MM.

To identify potential upstream regulators of PPRC1, Pearson correlation analyses were conducted between PPRC1 and other genes in two microarray datasets, and 14 positively co-expressed genes were obtained with *p* < .05 and R2 > 0.6 as the thresholds (Figure 5(H)). Motif enrichment analysis on these genes using ‘RcisTarget’ package identified 112 enriched motifs (Figure 5(I)). Among them, we focused on 39 motifs for which PPRC1 is a predicted target gene and visualized the regulatory network with their enriched genes (Figure 5(J)). Interestingly, key oncogenic transcription factors such as MYC and MAX were among the top annotated regulators, suggesting a direct regulatory link to PPRC1.

### 3.6. PPRC1 knockdown impairs the growth and survival of MM cells in vitro

**Figure 6.**
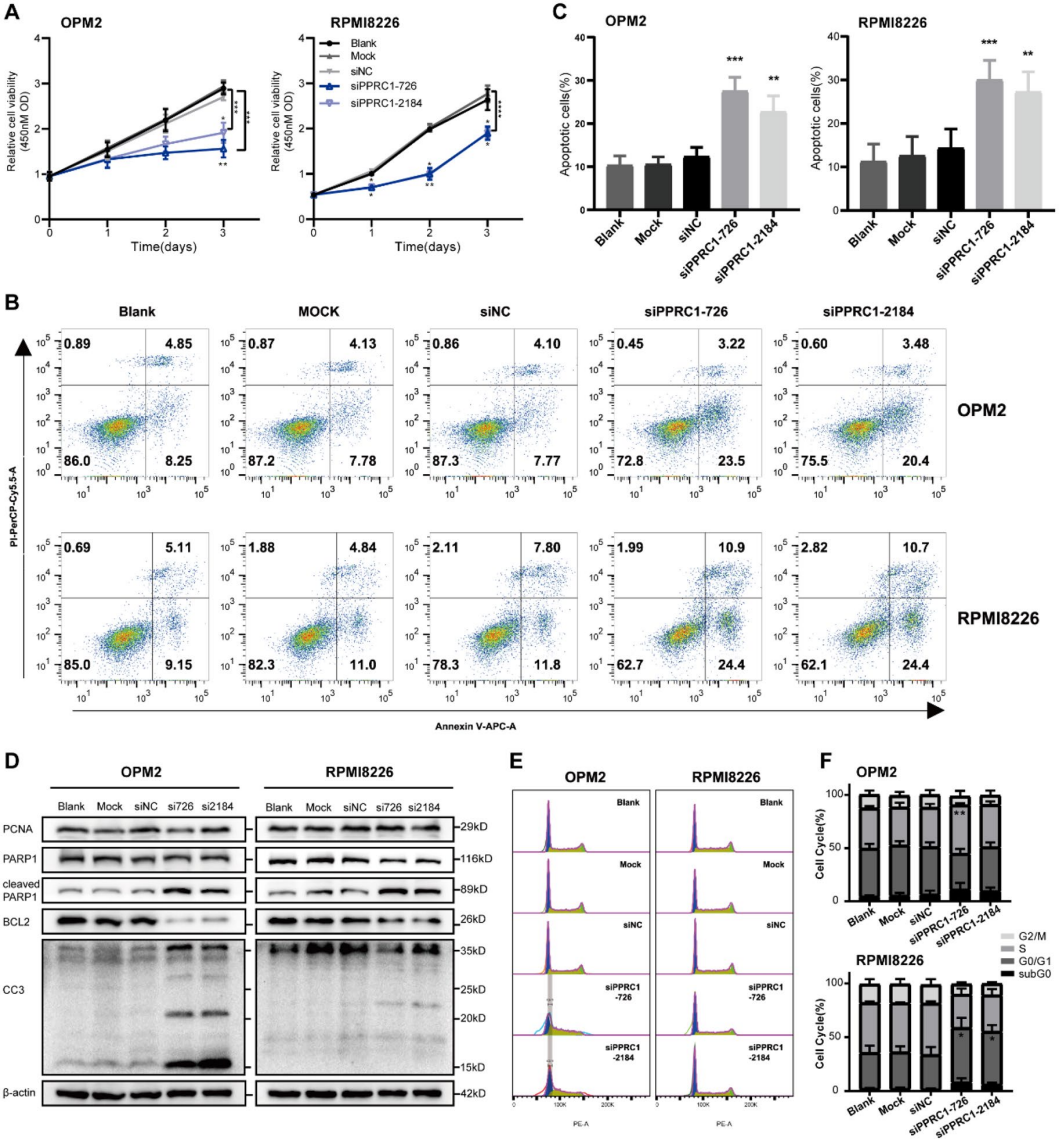
PPRC1 knockdown inhibits cell proliferation and induces cell apoptosis in MM. (A) Cell viability of control and PPRC1 knockdown OPM2 and RPMI8226 cells by CCK8 assay. PPRC1 knockdown inhibited MM cell proliferation. (B) The apoptosis rate of control and PPRC1 knockdown groups in OPM2 and RPMI8226 by Annexin V-PI flow cytometry analysis. Data shown are from one representative experiment of three replicates. (C) Quantitative results of OPM2 and RPMI8226 cells apoptosis after PPRC1 knockdown. (D) Immunoblots showing the levels of cell proliferation (PCNA) and apoptosis (PARP1, CC3, BCL2) related proteins in control and siPPRC1 transfection group of OPM2 and RPMI8226, β-actin is included as a loading control. (E) Cell cycle analysis of control and PPRC1 knockdown cells by PI staining flow cytometry analysis. Data shown are from one representative experiment of three replicates. (F) Quantitative results of OPM2 and RPMI8226 cells cell cycle distribution after PPRC1 knockdown. The statistical analysis shows mean values ± standard deviation of at least three independent experiments. Blank, Mock and siNC are the wild type, transfection reagent and non-target vector control groups, respectively. *p < .05, **p < .01, ***p < .001, ****p < .0001 compared to the siNC group.

To investigate the functional role of PPRC1 in MM, we silenced it expression in two HMCLs using specific small interfering RNA against PPRC1 (siPPRC1). Two independent siRNA sequences (siPPRC1-726 and siPPRC1-2184) effectively knocked down PPRC1 at both mRNA and protein levels in OPM2 and RPMI8226 cells (Figure S6) and were thus utilized in subsequent functional experiments. PPRC1 knockdown significantly inhibited the viability and proliferation of OPM2 and RPMI8226 cells versus controls (*p* < .05). This anti‑proliferative effect was dose‑dependent in OPM2 cells, supporting that PPRC1 is responsible for the growth inhibition (Figure 6(A)).

To assess the effect of PPRC1 inhibition on cell apoptosis, we used flow cytometry and detected an increased apoptosis rate in MM cells following PPRC1 silencing (Figure 6(B,C)). Western blot results showed that PPRC1 downregulation increased the expression of apoptosis-related proteins (cleaved caspase 3 and cleaved PARP1) but decreased the expression of antiapoptotic protein (BCL2) and proliferation-supporting protein (PCNA) (Figure 6(D)). PPRC1 knockdown also induced cell cycle arrest, with OPM2 cells showing S phase accumulation and RPMI8226 cells exhibited G0/G1 phase arrest (Figure 6(E,F)). Taken together, these data demonstrate that PPRC1 is involved in regulating the growth and survival of MM cells *in vitro*.

### 3.7. PPRC1 knockdown inhibits OXPHOS metabolism, induces mitochondrial lesions and activates the AMPK pathway

**Figure 7.**
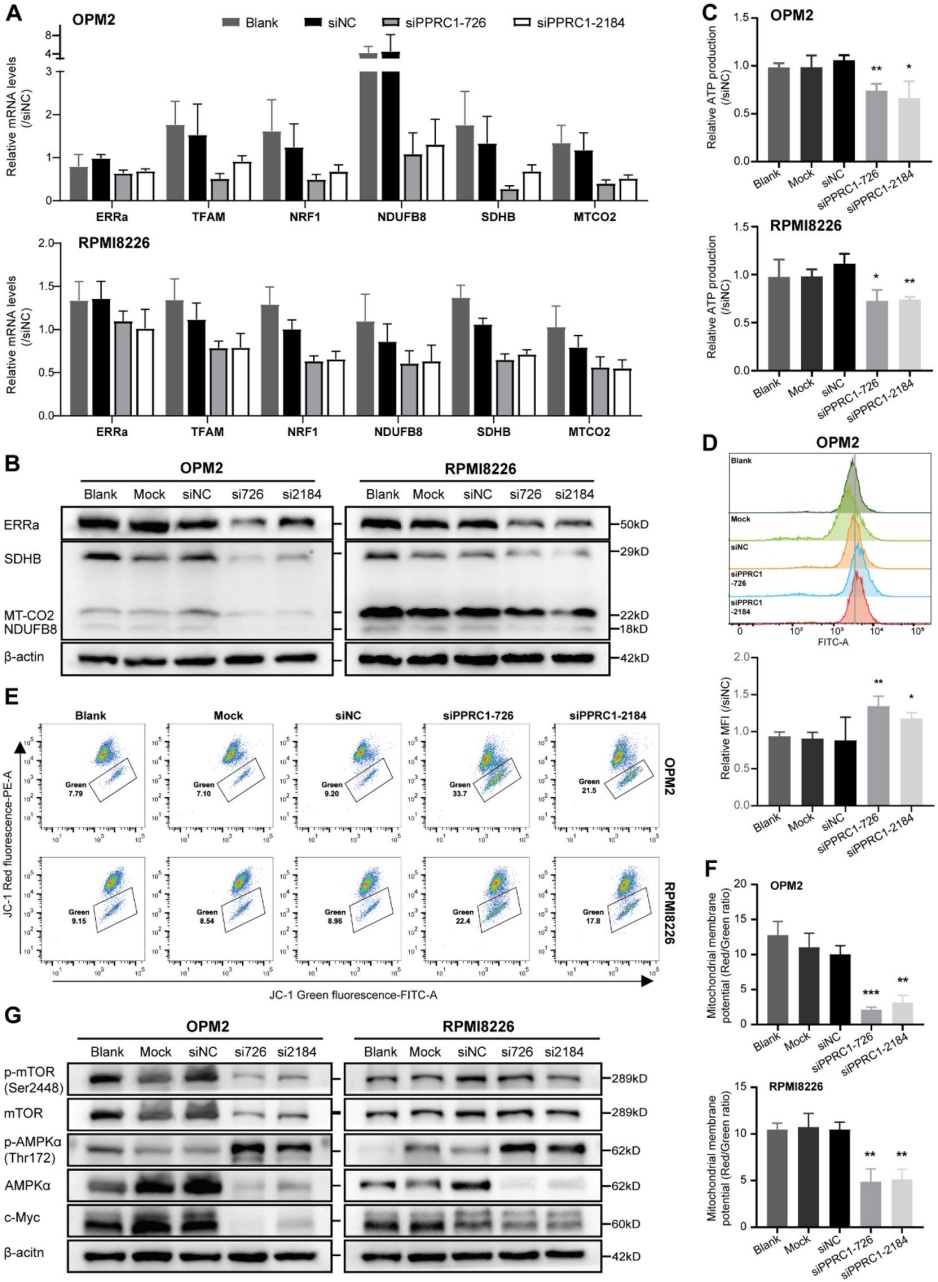
PPRC1 knockdown impairs OXPHOS metabolism in MM. (A) mRNA levels of OXPHOS metabolism related genes in control and PPRC1 knockdown OPM2 and RPMI8226 cells by RT-qPCR assay. (B) Protein expression changes of OXPHOS metabolism related molecules in OPM2 and RPMI8226 cells caused by PPRC1 silencing were detected by WB. (C) Barplots showing the change of intracellular ATP levels after PPRC1 knockdown in OPM2 (upper) and RIMI8226 (lower) cells by luminescent assay. (D) ROS level in the control and PPRC1 knockdown OPM2 cells was detected by DCFH-DA through flow cytometry. The lower barplot representing the quantitative results. (E) The mitochondrial potential was measured by JC-1 staining and flow cytometry to assess the degree of mitochondrial depolarization after PPRC1 knockdown in OPM2 and RPMI8226 cells. Data shown are from one representative experiment of three replicates. (F) Quantitative results of the ratio of JC-1 red to green fluorescence intensity in OPM2 (top) and RPMI8226 (bottom) cells. (G) The phosphorylation and protein expression level of mTOR, AMPKα and c-MYC in control and PPRC1 silencing OPM2 and RPMI8226 cells by WB. β-actin is included as a loading control. Data are shown as the mean ± standard deviation, *n* = 3–5. Student’s *t* test was used for statistical analysis, *p < .05, **p < .01, ***p < .001 compared to the siNC group.

We then investigated the underlying molecular mechanism by which PPRC1 promotes MM cell growth and proliferation. Given the bioinformatic link between PPRC1 and OXPHOS metabolism (Figure 3), we first measured the expression of key electron transport chain (ETC) molecules, including ERRα, TFAM, NRF1, NDUFB8, SDHB and MT-CO2. RT-qPCR showed that their mRNA levels were markedly decreased in PPRC1-knockdown OPM2 and RPMI8226 cells (Figure 7(A)). Further WB analysis confirmed diminished protein levels of ERRα, SDHB, NDUFB8 and MT-CO2 in PPRC1 silencing cells (Figure 7(B)), indicating coordinated suppression of respiratory subunits at both transcription and translation levels. Impaired ETC chains may affect OXPHOS metabolism, leading to insufficient energy production. Thus, we assessed the effect of PPRC1 inhibition on the total intracellular ATP content and reported that PPRC1 knockdown significantly reduced ATP levels in both OPM2 and RPMI8226 cells (Figure 7(C)).

Since OXPHOS also play a role in maintaining redox balance, we evaluated ROS levels using DCFH-DA fluorescent dye. Quantification revealed that ROS production increased to 1.5 times the control level in OPM2 cells (Figure 7(D)), indicating that oxidative damage was induced by PPRC1 depletion. Additionally, as shown in Figure 7(E,F), the ratio of red fluorescence from mitochondrial JC-1 aggregates to green fluorescence from cytoplasmic JC-1 monomers significantly decreased from 10.03 ± 1.22 (siNC group) to 2.13 ± 0.35 (siPPRC1-726 group, *p* < .001) and 3.16 ± 1.01 (siPPRC1-2184 group, *p* < .01) in OPM2 cells. In the RPMI8226 cells, the ratio decreased from 10.5 ± 0.75 (siNC group) to 4.88 ± 1.37 (siPPRC1-726 group, *p* < . 01) and 5.13 ± 1.07 (siPPRC1-2184 group, *p* < .01), indicating mitochondrial depolarization and dysfunction following PPRC1 knockdown. In addition, our results revealed that the phosphorylation level of AMPKα at Ser172 significantly increased with PPRC1 knockdown in both OPM2 and RPMI8226 cells, indicating that AMPK signalling was activated. Moreover, phosphorylation of mTOR at Ser2448 markedly decreased with PPRC1 depletion. The expression of proto-oncogene c-MYC, which is closely related to cancer metabolism, was also reduced following PPRC1 knockdown (Figure 7(G)). Taken together, our results confirm that PPRC1 downregulation induces apoptosis by impairing OXPHOS metabolism, promoting ROS accumulation and activating AMPK signalling.

## 4. Discussion

An increasing body of research has highlighted the role of PGC-1s genes, primarily PPARGC1A and PPARGC1B, in regulating tumorigenesis and cancer development through their influence on metabolism. However, the role of PPRC1 in tumours has been less explored [8]. This study provides the first comprehensive evidence that PPRC1 is a clinically relevant biomarker and functional oncogenic regulator in multiple myeloma. By integrating large-scale transcriptomic datasets with *in vitro* experiments validation, we demonstrated that PPRC1 is upregulated in MM, with its overexpression linked to malignant clinicopathological characteristics and poor prognosis. PPRC1 knockdown impeded cell growth and survival *in vitro*. Mechanistically, PPRC1 supports mitochondrial OXPHOS metabolism, regulating energy production and redox homeostasis in MM cells.

PPRC1, the third member of the PGC-1s transcriptional coactivator family, was first identified as a factor that activates mitochondrial biogenesis, partially through direct interactions with transcription factors related to the ETC in response to cellular proliferative signals [17]. Compared with research on its two family members, research on the pathological effects of PPRC1 in humans remains limited. Recent studies have linked PPRC1 to intervertebral disc degeneration and sepsis [18,19]. Moreover, by reducing mitochondrial biogenesis, PPRC1 has been reported to be associated with anti-inflammatory responses in stimulated Th17 cells [20]. In cancer, PPRC1-mediated mitochondrial biogenesis is a downstream target through which eugenol kills leukaemia cells [21]. Xingqiu Ruan et al. proposed that PPRC1 is a promising onco-promoting biomarker that potentially influences the TME in ovarian cancer and liver cancer [22]. In glioblastoma, PPRC1 was reported to be a mediator of the COL5A2-ESM2 axis to promote tumour metastasis [23]. In our study, high PPRC1 expression was observed in MM through public datasets and was confirmed in our local patient samples at both mRNA and protein levels. Correlation analyses revealed that increased expression of PPRC1 was associated with advanced disease signatures, including advanced ISS or RISS stages, increased TMB, the presence of CAs, high-risk gene expression profiles (GEP), proliferation subgroup and inferior survival outcomes. Notably, a significant correlation between PPRC1 expression and high-risk CAs was verified in our local cohort. Moreover, multivariate Cox analysis indicated that the PPRC1 is an independent prognostic factor. Our *in vitro* experiments demonstrated that PPRC1 inhibition resulted in cytostatic and cytotoxic effects on MM cells, supporting the notion that PPRC1 is a potential oncogene influencing MM pathogenesis and further study of the specific molecular mechanisms involved is warranted.

Early diagnosis and stratified therapeutic strategies are highly important for improving the prognosis of MM patients. The RISS is widely applied in the risk stratification of MM, with good applicability in clinical practice. However, current definitions of high-risk MM also present challenges, potentially leading to suboptimal therapeutic regimens and imprecise prognostic assessments for certain patients [15]. It is common for disease risk stratification systems to exhibit such limitations due to advances in diagnosis and treatment, highlighting the need for ongoing refinement of risk assessment in patients with MM. Since our research revealed that PPRC1 is an independent risk factor for MM patients, we aimed to incorporate it into a nomogram model alongside the RISS. As anticipated, compared with the RISS alone, the nomogram model showed satisfactory survival prediction capability for MM patients, offering superior clinical net benefit and predictive ability. Hence, PPRC1 incorporation may enhance the efficacy of risk stratification for MM patients.

OXPHOS metabolism has been recognized as a critical factor in MM progression. Previous studies have shown that MM cells from patients with poor prognostic signatures show significantly enriched OXPHOS expression [24,25]. Additionally, sensitivity to proteasome and BCL2 inhibitors in MM cells has been linked to OXPHOS metabolism [26–28]. Various molecules have been showed to regulate OXPHOS in MM, further emphasizing the importance of this metabolic pathway. For instance, both IL-32 signalling and CK1δ/CK1ε signalling have been reported to support MM cell survival by sustaining mitochondrial OXPHOS metabolism [29,30]. CD38 and the CXCR4/CXCL12 axis drive mitochondrial transfer between bone marrow stromal cells and MM cells to support OXPHOS metabolism and promote bioenergetic plasticity [31,32]. Our study suggested an oncogenic role of PPRC1-induced OXPHOS metabolism in MM. GSEA and scMetabolism analyses revealed a positive correlation between PPRC1 expression and OXPHOS metabolism. Besides, siRNA-mediated PPRC1 knockdown in MM cells impaired OXPHOS complex, resulting in reduced ATP production and MMP, along with elevated ROS levels and AMPK phosphorylation, highlighting PPRC1 as a promising target for energy metabolism in MM.

Notably, the expression of ERRα, TFAM and NRF1, which are well-known transcription factors involved in OXPHOS metabolism [33,34], decreased in PPRC1- silencing cells. While our motif enrichment analysis revealed that ERRα (annotated for metacluster_90.4 motif) might be the upstream transcriptional regulator of PPRC1, TFAM and ERRα itself (Figure S7), no evidence supported a direct transcriptional regulation of these genes by PPRC1. It has been established that ERRα regulates its own expression by interacting with PPARGC1A, a mechanism corroborated by the presence of a functional ERRα binding site in its proximal promoter region [35,36]. Given that ERRα is a documented binding partner of PPRC1 and their complex is implicated in direct regulation of NRF1 [37,38], we proposed that PPRC1 and ERRα forms a functional regulatory axis. Consequently, knockdown of PPRC1 is expected to attenuate ERRα transcriptional activity, leading to suppressed expression of OXPHOS-related genes as well as ERRα itself. On the other hand, many studies have reported bidirectional communication between the nucleus and mitochondria. Specifically, mitochondrial dysfunction can send feedback signals to the nucleus and influence nuclear gene expression [39,40]. In our study, mitochondria damage occurred in cells with PPRC1 knockdown, which might also influence the expression of OXPHOS related genes through a mito-nuclear feedback loop. Further study is necessary to elucidate the specific mechanism involved in MM.

Another intriguing finding from our study was the reduced expression of c-MYC following PPRC1 knockdown. c-MYC is known to promote anabolic metabolism by regulating metabolic gene expression, which is involved mainly in glycolysis and glutaminolysis in cancer[41,42]. Our motif analysis in MM provides further support for the c-MYC-PPRC1 interaction (Figure 5(I)), which is known to reprogram gene expression during cellular dysfunction [43,44]. While direct evidence for PPRC1 acting on c-MYC is lacking, c-MYC is established as a target of energy stress and AMPK signalling. This is supported by studies showing that sodium salicylate-induced AMPK activation promotes c-MYC phosphorylation, ubiquitination and nuclear depletion [45], and that energy stress-induced lncRNA FILNC1 suppresses c-MYC expression by sequestering AUF1 [46]. Notably, we demonstrate that PPRC1 knockdown triggers energy stress and AMPK activation. Thus, c-MYC downregulation upon PPRC1 loss is likely an indirect, AMPK-mediated effect in MM cells. Elucidating this PPRC1/energy stress/c-MYC axis in MM could inform the development of alternative c-MYC-targeted therapies.

In addition to tumour-intrinsic effects, our study uncovered a negative correlation between PPRC1 expression and immune cell infiltration in MM microenvironment. This is of particular relevance given the established role of the TME as a critical facilitator of tumorigenesis and disease progression [47]. Notably, our TME analysis indicated a specifically lower proportion of neutrophils/granulocytes in PPRC1 high patients, aligning with established evidence linking low neutrophil abundance to unfavourable prognosis in MM [48,49]. Cell subcluster analysis for scRNA-seq data of MM BMMNC also revealed remarkable difference of cell subtype abundance and T cell function between PPRC1 expression group. These findings suggest that PPRC1 may contribute to immune compromised and tumour progression, although the precise mechanisms require further investigation. The relationship between tumour metabolism and immune suppression is increasingly recognized, and our results place PPRC1 at the intersection of these two processes in MM.

Several limitations of this study should be acknowledged. First, sample size of the local cohort was relatively small, limited the statistical power for subgroup analyses in MM. Future studies should aim to expand the NDMM sample size, as well as RRMM and PCL samples, to increase the evidence for its prognosis and progression promoting role in MM. Second, although *in vitro* experiments demonstrated a clear functional role for PPRC1, *in vivo* xenograft mouse model exploring whether PPRC1 knockdown or even knockout can retard tumour growth is necessary for future research to confirm its oncogenic effects in MM. Third, mechanistic studies such as rescue experiments (e.g. overexpression of PPRC1 or its downstream OXPHOS related transcription factors) and protein interaction assays are required to establish evidence for their direct or indirect interaction in MM. Finally, the upstream regulatory network connecting MM driver factors and PPRC1 dysregulation requires further investigation.

## 5. Conclusions

Our study identifies PPRC1 as a novel prognostic biomarker and metabolic regulator in MM for the first time. PPRC1 is highly expressed and associated with poor prognosis in MM patients. The independence and predictive efficiency of PPRC1 for MM prognosis were substantiated through our analyses. Functionally, the molecular mechanisms through which PPRC1 promotes MM involve multiple signalling pathways, including OXPHOS, gene expression, cell cycle and immune suppression. Our *in vitro* experiments demonstrated that PPRC1 knockdown disrupts OXPHOS metabolism, leading to both cytostatic and cytotoxic effects in MM cells. These findings provide a strong rationale for further exploration of PPRC1 as a therapeutic target and support its potential clinical utility in MM management.

## Supplementary Material

Supplementary files_clean.docx

## Data Availability

The experimental data will be made available on reasonable request. The public datasets analysed in this study are available in The Cancer Genome Atlas (https://portal.gdc.cancer.gov/), GEO database (http://www.ncbi.nlm.nih.gov/geo/) and ArrayExpress database (https://www.ebi.ac.uk/arrayexpress/).
