# Supplementary material for: PPRC1 is a prognostic biomarker and key regulator of mitochondrial oxidative phosphorylation in multiple myeloma
Source: Ann Med. 2026 Mar 11;58(1):2639658. doi: 10.1080/07853890.2026.2639658 (PMC12983811; doi:10.1080/07853890.2026.2639658)
Supplement: Supplementary files_clean.docx [file IANN_A_2639658_SM1977.docx]

**PPRC1 is a prognostic biomarker and key regulator of mitochondrial oxidative phosphorylation in multiple myeloma**

**Yilin Liu^1†^, Siqi Yan^1†^, Xuemei Shu^1^, Qiang Xu^1^, Min Zhang^1^, Yuxiang Wang^1^, Dawei Wang^2*^, Tao Guo^1,3*^**

^1^Institute of Hematology, Union Hospital, Tongji Medical College, Huazhong University of Science and Technology, Wuhan 430022, China

^2^Shanghai Institute of Hematology, State Key Laboratory of Medical Genomics, National Research Center for Translational Medicine at Shanghai, Ruijin Hospital Affiliated to Shanghai Jiao Tong University, Shanghai 200025, China

^3^Collaborative Innovation Center of Hematology, Huazhong University of Science and Technology, Wuhan 430022, China

**^†^Equal contribution**

These authors have contributed equally to this work;

**^*^Corresponding author**

E-mail: [wangdawei@shsmu.edu.cn](mailto:wangdawei@shsmu.edu.cn) (D.W.); [guotao1968@163.com](mailto:guotao1968@163.com) (T. G.)

**Supplementary methods**

1. **Public data acquisition and preprocessing**

This study utilized 18 multiple myeloma (MM) datasets and one normal tissue dataset from Gene Expression Omnibus (GEO) database (https://www.ncbi.nlm.nih.gov/geo/), including GSE5900, GSE6477, GSE16122, GSE47552, GSE16558, GSE116294, GSE205447, GSE39754, GSE153380, GSE193531, GSE175384, GSE24080, GSE136400 (including GSE136324 and GSE136337), GSE57317, GSE26863, GSE44745, GSE21304, GSE223060 and GSE120795. Additionally, E-MTAB-363 and E-TABM-1138 datasets were obtained from ArrayExpress (https://www.ebi.ac.uk/biostudies/arrayexpress/). The mRNA expression, methylation, genome variation data, and clinical information were directly downloaded from the respective databases. Both E-MTAB-363 and GSE5900 utilized the GPL570 platform, while GSE47552 and GSE16558 using GPL6244, GSE6477 and GSE16122 using GPL96 platform. These datasets were combined into three larger datasets after removing batch effects using the "sva" R package. The single cell RNA sequencing dataset, GSE193531 and GSE223060, was analyzed according to the previous article [1, 2]. RNA sequencing and clinical data from the MMRF-COMPASS project were downloaded from the GDC Data Portal (https://portal.gdc.cancer.gov/), and transcript per million (TPM) was calculated for gene quantification. Samples lacking sufficient clinical data were excluded from further prognostic evaluation analyses. Expression data of PGC-1s in pan-cancer tissues, including data from GDC MMRF-COMMPASS and GDC Pan-Cancer (PANCAN) datasets, were download from UCSC Xena database (https://xenabrowser.net/datapages/). The mRNA expression, genome variation data and clinic data in the Cancer Cell Line Encyclopedia (CCLE) were obtained from the DEPMAP database (https://depmap.org/portal/).

1. **Independence analysis and nomogram model analysis**

The classical prognostic factors of MM and PPRC1 were explored for independent prognostic factors via univariate and multivariate Cox regression analyses. Factors with a p < 0.05 in the univariate analysis were selected to execute the multivariate analysis. To evaluate the predictive power of PPRC1 and RISS factors, a nomogram was constructed by the “rms” R package in GSE136337 dataset, and their performance in predicting OS was assessed by the concordance index (C-index) and calibration curve. The time-dependent ROC and AUC of RISS and PPRC1 alone or combination were analyzed to compare their predictive capacity.

1. **PPRC1-related genes and pathway enrichment analysis**

Based on the trisection values of PPRC1 mRNA expression in GSE136337, GSE24080, and MMRF datasets, samples in the first and third trisections were categorized into PPRC1 low and high expression groups, respectively. The “limma” and “DESeq2” R package were employed to identify differentially expressed genes (DEGs) for microarray and RNA-seq datasets, respectively, with a threshold set at a fold change of > 1.5 and an adjusted p-value < 0.05. Gene Ontology (GO) or Hallmark enrichment analyses were conducted to determine the biological functions and signaling pathways associated with PPRC1.

1. **Regulatory Network Analysis**

Our study used the “RcisTarget” R package to identify significantly over-represented DNA motifs and annotate motif to transcription factors. All calculations performed using the RcisTarget were based on motifs. The normalized enrichment score (NES) of a motif depends on the total number of motifs in the database. Additionally, the NES for each motif was calculated based on the distribution of the AUCs for all motifs in the gene set. The hg38_10kbp_up_10kbp_down_full_tx_v10_clust.genes_vs _motifs.rankings.feather and motifAnnotations_hgnc was used for the Gene-motif rankings database and annotation reference[3].

Pearson correlation analysis was used to find PPRC1 positive co-expressed genes in GSE136337 and GSE24080, with |R2| > 0.6 and *p* < 0.05 considered as significant threshold. Significant genes presented in both datasets were include into the motif enrichment analysis.

1. **Single‐cell RNA sequencing data analysis**

We use the Seurat (version 5.1.0) R package to preprocess the GSE193531 scRNA-seq data. Quality control criteria were as followed: cells with 200 < nFeature_RNA < 4000 and nCount_RNA <50000 and mitochondrial gene percent < 15 were included into analysis. In dimensionality reduction analysis, the resolution and dimensions were set as 0.8 and 30, respectively. Scissor was used to identify bulk phenotype-associated cell subsets from scRNA-seq data. PPRC1 low and high expression patients identified in GSE136337 were treated as two different phenotypes and “binomial” was chosen as the response type for the regression model. We choose an alpha value of 0.9 according to the limitation of total Scissor selected cells (default 20%) in the single-cell data. The Reliability.Test function was used to perform a reliability significance test. FindMarkers function facilitated the identification of DEGs across each Scissor subpopulations, |avg.logFC|>0.2 and p<0.05 were set as significant threshold. scMetabolism package was employed to quantify the metabolic activity within distinct subtypes of Scissor cells at the single cell resolution. We used “AUC” method and “KEGG” pathway to evaluate cell metabolism.

GSE223060 dataset collected patient samples from three 3 independent studies. The MMRF cohort include 18 CD138- BMMNC samples of NDMM patients, 16 of them have match CD138+ samples bulk RNA-sequencing data in GDC MMRF dataset. Quality control criteria were as followed: cells with 200 < nFeature_RNA < 5000 and 1000< nCount_RNA <50000 and mitochondrial gene percent < 20 were included into analysis. We used harmony to merged cells from multiple samples, and 0.8 and 30 were set as resolution and dimensions respectively in dimensionality reduction analysis. “FindAllMarker” were used to obtain cell type specific markers. Erythroid cells (HBA1, HBA2, HBB) were exclude from further analyses. The classic cell type markers were shown in Fig. S4B. The marker genes used for cell type subclustering analysis were a collection from these 3 articles [4-6].

1. **Tumor immune infiltration analysis**

Consistent with DEG analysis, 426 patients with CD138+ cell samples were grouped based on the trisection values of PPRC1 mRNA level in GSE136337 dataset. A total of 326 patients with match whole bone marrow (WBM) samples before treatment were screened to perform tumor immune infiltration (TIL) analysis in GSE136324 dataset, including 113, 109, and 104 patients in the high, middle, and low expression groups, respectively. ESTIMATE algorithm was used to evaluate the overall immune infiltration by the “estimate” R package. Tumor Immune Dysfunction and Exclusion (TIDE) was performed to evaluate the potential of tumor immune escape in the TIDE website (http://tide.dfci.harvard.edu/). To comprehensively investigate the relative abundance of the infiltrating immune cells in the WBM samples of MM patients, we used xCell, single sample gene set enrichment analysis (ssGSEA) [7], and CIBERSORT [8] in R. Other methods including CIBERSORT_ABS, quanTIseq, MCP-counter, and EPIC were also conducted via the TIMER webserver (http://timer.comp-genomics.org/). The association between the expression of PPRC1 and immune-related molecules was investigated using Spearman correlation analysis, and *p* < 0.05 was set as significant threshold. The “ComplexHeatmap” and “circlize” R packages were used to draw circle heatmap. Patients (n = 243) with match WBM samples before and after treatment were chosen for longitudinal comparative analysis in GSE136324.

1. **Gene variation analysis**

Mutation status was acquired from VarScan2 MAF files for simple nucleotide variation in the MMRF datasets, which were accessed through the GDC Data Portal. Tumor mutation burden (TMB) was calculated based on the total number of non-silent mutations in specimens. MAF files for the MMRC dataset were downloaded from cBioportal (https://www.cbioportal. org/). The “maftools” R package was utilized to analyze the somatic mutations and mutational signatures across samples. GISTIC was used to calculate chromosomal copy number aberrations (CNA) via the GenePattern platform (https://cloud.genepattern.org/).

**Supplementary tables and figures**

**Table S1** Primary antibodies used in WB assay.

| Reagent | source | identifier |
| --- | --- | --- |
| PCNA | AntGene | ANT333 |
| PARP1 | Zen Bioscience | R25279 |
| Cleaved-PARP1 | Zen Bioscience | R380374 |
| BCL2 | Abcam | ab32124 |
| Cleaved Caspase 3 | Cell Signaling Technology | 9664S |
| Total OXPHOS Human WB Antibody Cocktail | Abcam | ab110411 |
| ERRα | Zen Bioscience | R381173 |
| p-mTOR (Ser2448) | Cell Signaling Technology | 5536T |
| mTOR | Zen Bioscience | 380411 |
| p-AMPKα(Thr172) | Cell Signaling Technology | 2535T |
| AMPKα | Cell Signaling Technology | 5832T |
| c-Myc | Cell Signaling Technology | 9402S |
| β-actin | AntGene | ANT422 |

**Table S2** RT-qPCR primer and siRNA sequences.

| Gene | Forward/sense (5’->3’) | Reverse/antisense (5’-> 3’) |
| --- | --- | --- |
| ACTB | CATGTACGTTGCTATCCAGGC | CTCCTTAATGTCACGCACGAT |
| PPRC1 | CAGTGGTTGGGGAAGTCGAAG | GCCGAGAGAGACTGACAAAGC |
| ERRa | CCTGAGAAGCTCTATGCCATGC | CAAAGAGGTCACAGAGGGTAGC |
| TFAM | GCGGGTTCCAGTTGTGATTG | CACATGCTTCGGAGAAACGC |
| NRF1 | TCTCCACGTCTTGCTCAACC | ATCCATGCTCTGCTACTGGG |
| NDUFB8 | GTTGAACTGGGGTGAACCGA | AGGAAACCGAAGAGCTGCAT |
| SDHB | CTAGCTTGCACCCGAAGGAT | GTCTCCGTTCCACCAGTAGC |
| MTCO2 | CTGCGACTCCTTGACGTTGA | GGTCGTGTAGCGGTGAAAGT |
| siPPRC1-1620 | GGAGAGAAGUGCUGGACAATT | UUGUCCAGCACUUCUCUCCTT |
| siPPRC1-726 | GCGCAGUGAUGGAGAACAATT | UUCUUCUCCAUCACUGCGCTT |
| siPPRC1-2184 | GGUUGUGGAUUCUCUGAAATT | UUUCAGAGAAUCCACAACCTT |
| Negative control | UUCUCCGAACGUGUCACGUTT | ACGUGACACGUUCGGAGAATT |

**Table S3** Univariate Cox regression analysis of PPRC1 in MM

| Dataset | Number | Coefficient | HR (95%CI) | P-value | Outcome | Disease |
| --- | --- | --- | --- | --- | --- | --- |
| GSE136337 | 426 | 0.381 | 1.464(1.097-1.952) | 0.0095 | OS | NDMM |
| GSE136337 | 426 | 0.589 | 1.802(1.39~2.32) | 7.13E-06 | PFS | NDMM |
| GSE24080 | 551 | 0.442 | 1.556(1.134~2.135) | 0.0061 | OS | NDMM |
| GSE24080 | 551 | 0.314 | 1.369(1.049~1.785) | 0.0206 | EFS | NDMM |
| MMRF | 744 | 0.252 | 1.287(0.943~1.755) | 0.1120 | OS | NDMM |
| MMRF | 744 | 0.483 | 1.621(1.054~2.493) | 0.0278 | DSS | NDMM |
| MMRF | 80 | 0.984 | 2.676(1.65~4.341) | 6.65E-05 | OS | RRMM |
| GSE57317 | 55 | 1.118 | 3.058(1.13~8.274) | 0.0277 | OS | RRMM |

Note: Abbreviations: OS, overall survival; PFS, progression-free survival; EFS, event-free survival; DSS, disease-specific survival.


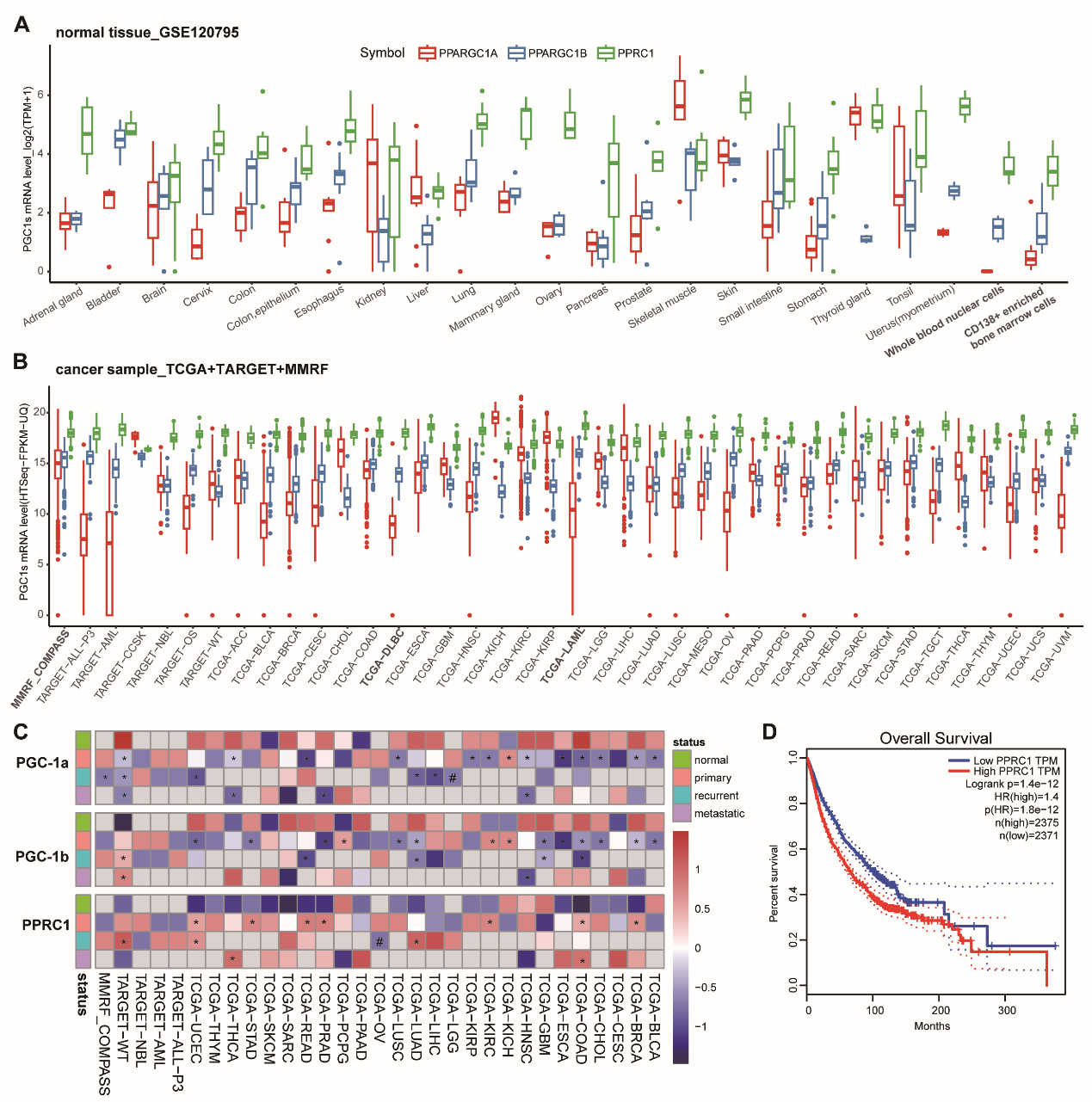


**Fig. S1** **Overview of PGC-1s in pan normal and cancer tissues.** Boxplot showing the mRNA level of PGC-1s across 23 types of normal tissues in GSE120795 from GEO (A), 40 types of cancer tissues in GDC pan-cancer and GDC MMRF-COMMPASS datasets from USCS Xena (B). (C) Heatmap showing the mean expression of PGC-1s in normal tissue and primary, recurrent or metastatic tumor tissue. **p*<0.05 compare to normal, #*p*<0.05 compare to primary. (D) Kaplan-Meier survival curves of OS in pan cancer patients based on PPRC1 low (lower quartile) and PPRC1 high (upper quartile) expression group by GEPIA2 websites.


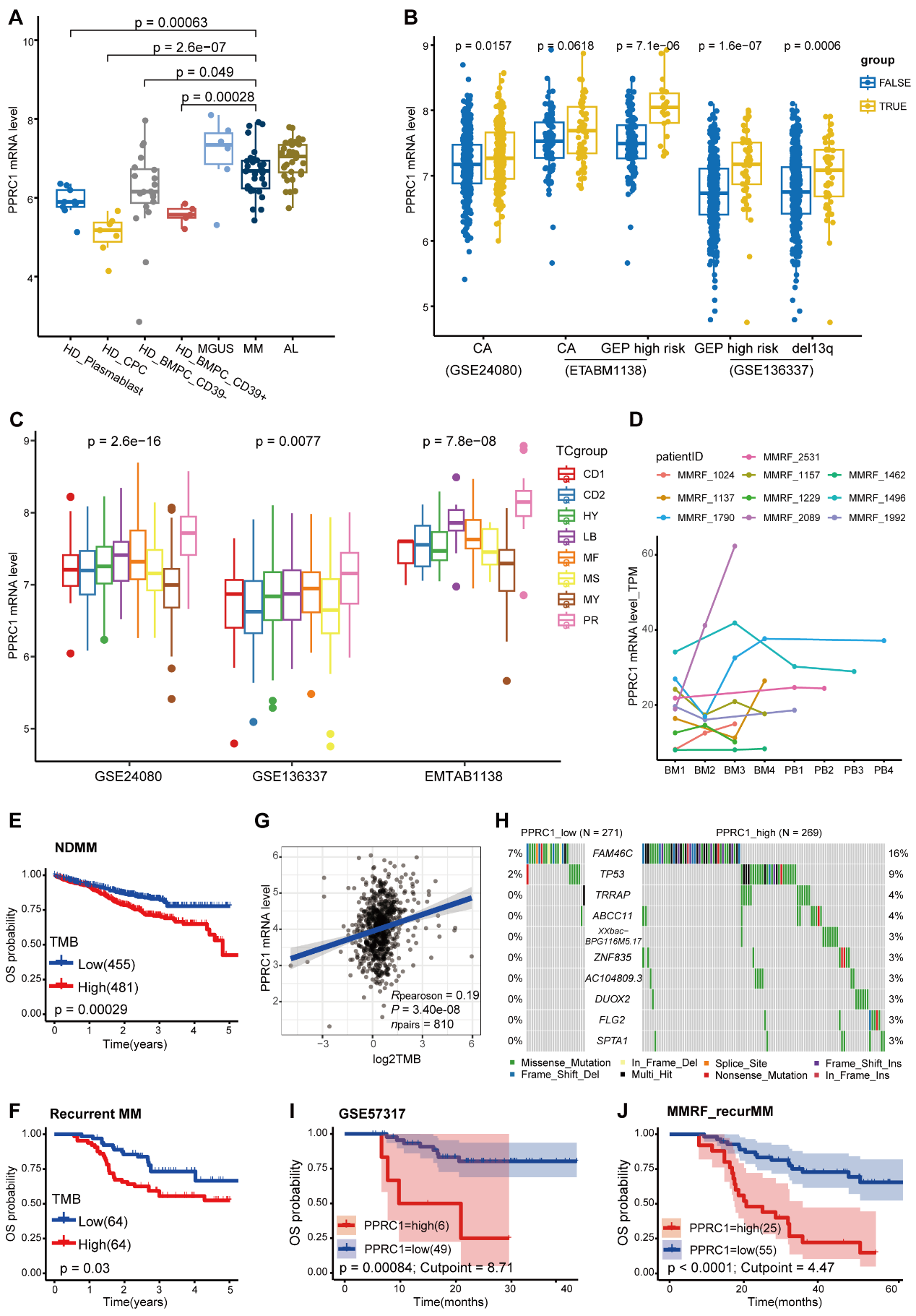


**Fig. S2** **The expression and prognostic significance of PPRC1 in MM.** Comparison of PPRC1 mRNA level in different developmental stages or tissue sources of plasma cell in GSE175384 (A), distinct genetic subgroups (B), and translocation and CCND1 (TC) molecular subgroups of MM patients in several datasets (C). PPRC1 mRNA level was higher in patients with cytogenetics abnormality (CA) or high risk GEP signatures, and proliferation (PR) subgroup compared to others. (D) PPRC1 expression level in the longitudinal samples of MM patients from MMRF. BM and PB means samples source from bone marrow and peripheral blood, respectively. The latter numbers from 1 to 4 indicate primary, first relapse, second relapse, and third relapse. Kaplan-Meier survival curves of OS of MM patients with primary (E) or recurrent (F) disease states based on a high or low TMB level stratified by the median value in MMRF. (G) Scatterplot showing the positive correlation between TMB and PPRC1 mRNA level in MMRF. (H) Oncoplot showing the top 10 most different mutated genes between PPRC1 high and low expression groups in MMRF. Kaplan-Meier analysis of OS according to PPRC1 mRNA level of RRMM patients in GSE57317 (I) and MMRF (J). The grouping threshold for PPRC1 was the optimal cutpoint calculated by the “survminer” R package. HD, healthy donor; CPC, circulating plasma cell; BMPC, bone marrow plasma cell; MGUS, monoclonal gammopathy of undetermined significance; MM, multiple myeloma; AL, amyloidosis; CA, cytogenetics abnormality; GEP, gene expression profile; TC group, translocation/ cyclin D molecular group; NDMM, newly diagnosed MM; TMB, tumor mutation burden; recurMM, recurrent MM.


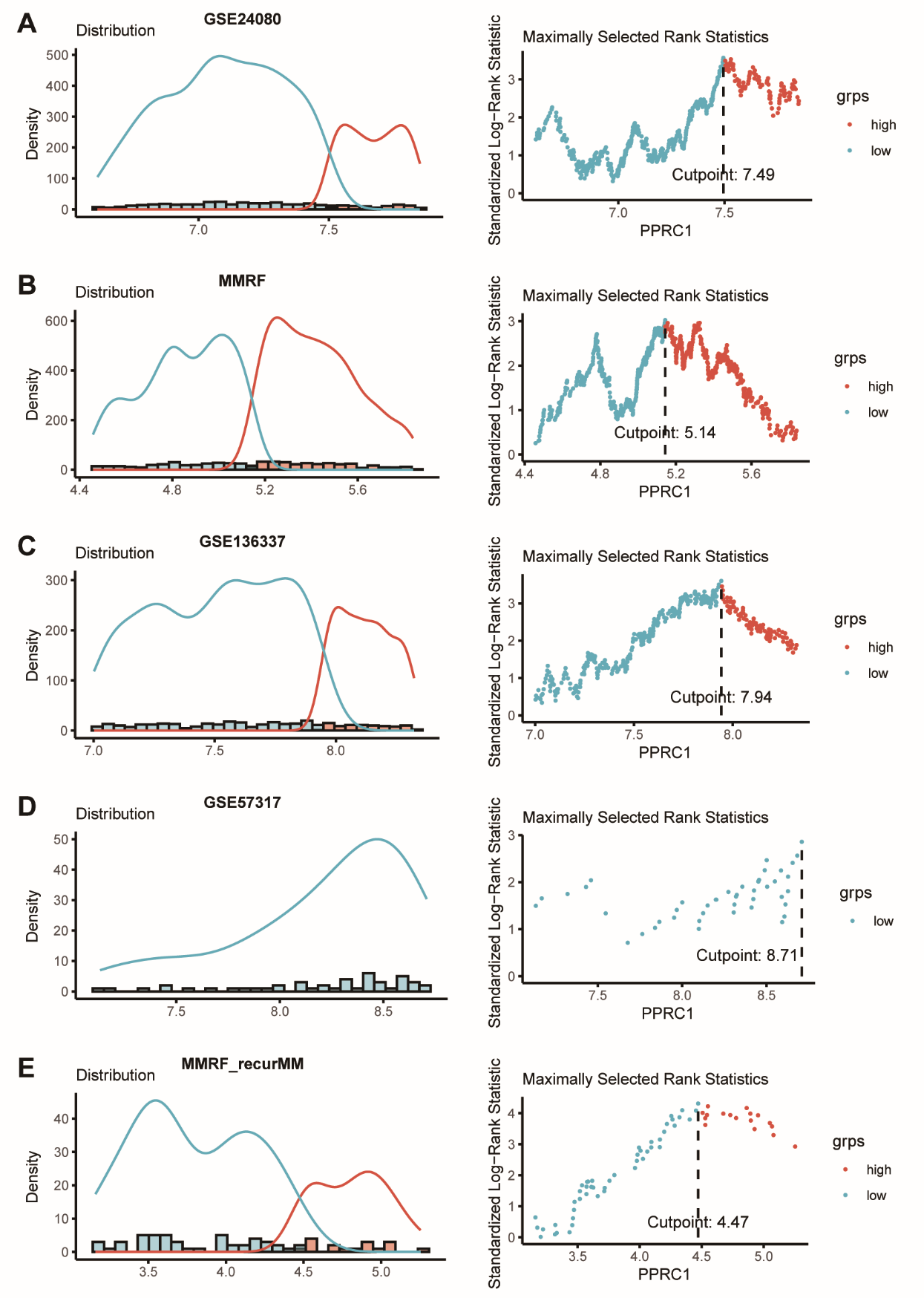


**Fig. S3** **Visualization of maxstat results for Kaplan-Meier survival analysis in MM.** The maxstat results for optimal cutpoint of PPRC1 gene in GSE24080 (A), MMRF (B), GSE136337 (C), GSE57317 (D), and MMRF_recurMM (E) datasets.


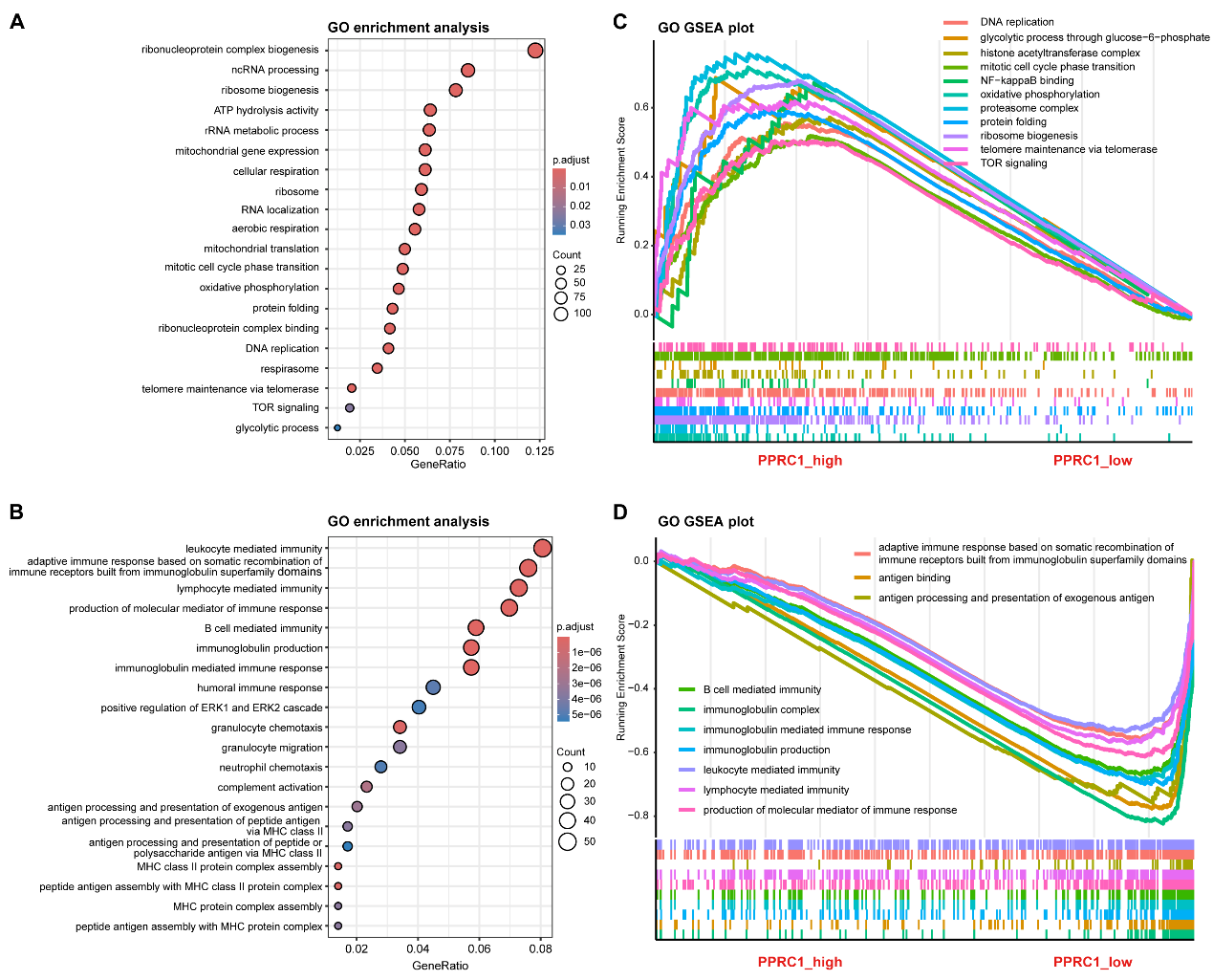


**Fig. S4** **PPRC1-related genes enrichment analysis in GSE24080 and MMRF.** Bubble plot of Gene Ontology (GO) functional enrichment analysis of PPRC1-related DEGs in GSE24080 (A) and MMRF (B) datasets. GSEA plot exhibited top GO terms significantly enriched in PPRC1 high expression group in GSE24080 (C) and MMRF (D) datasets. The gene number range was set to 10 to 500.


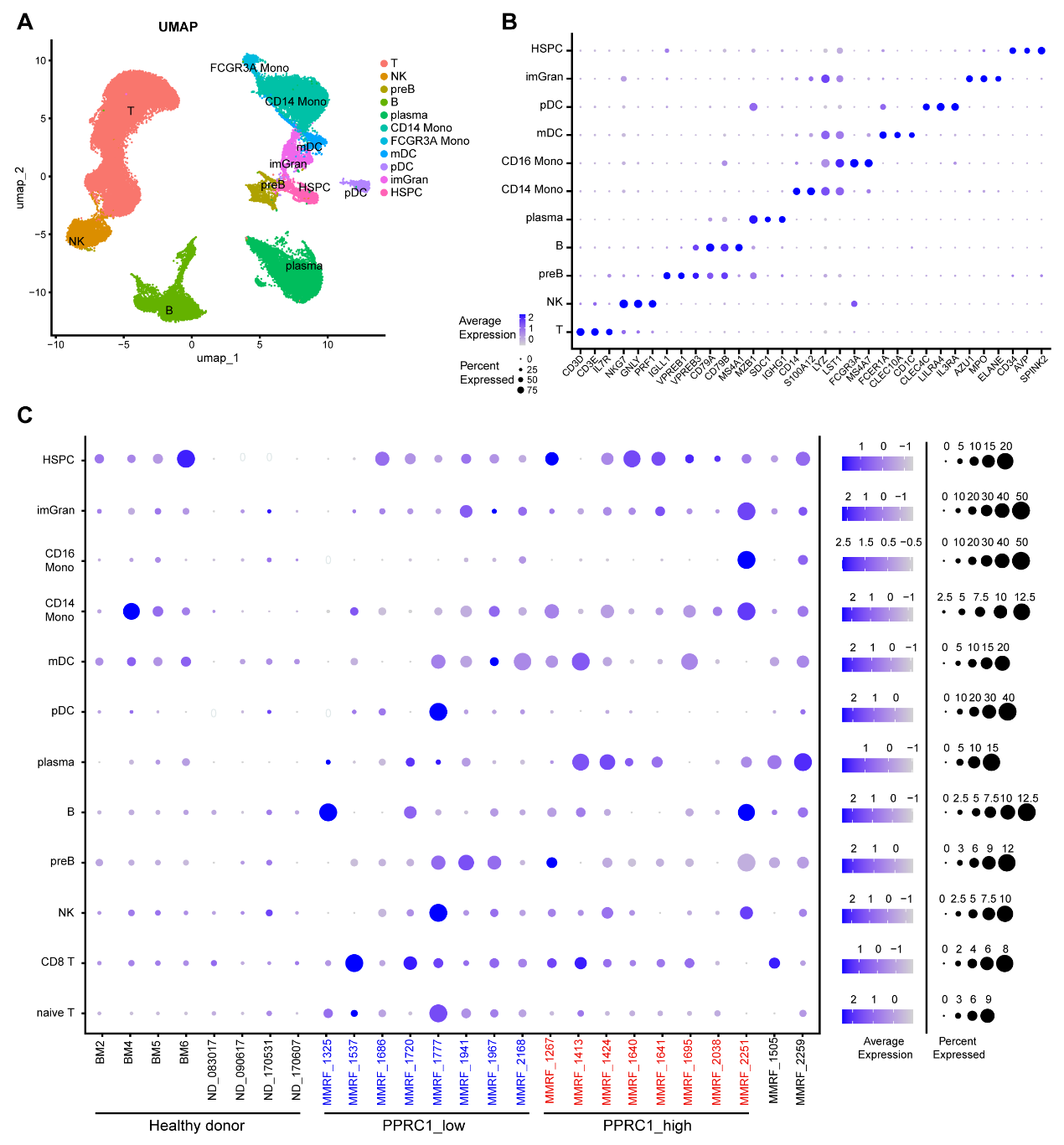


**Fig. S5** **TME analysis in MMRF scRNA-sequencing dataset.** (A) UMAP plot showing 11 cell clusters identified in the BM samples in GSE223060_MMRF cohort. (B) Dotplot of cell-type-specific marker genes in each cell types. Dot intensity (from grey to blue) represents the average expression value, whereas dot size represents the proportion of cells expressing the genes. (C) Dotplot showing expression of PPRC1 in each cell types for healthy donor and PPRC1 high and low patients. preB, precusor B cell; Mono, monocyte; mDC, myeloid dendritic cell; pDC, plasmacytoid dendritic cell; imGran, immature granulocyte; HSPC, hematopoietic stem and progenitor cell.


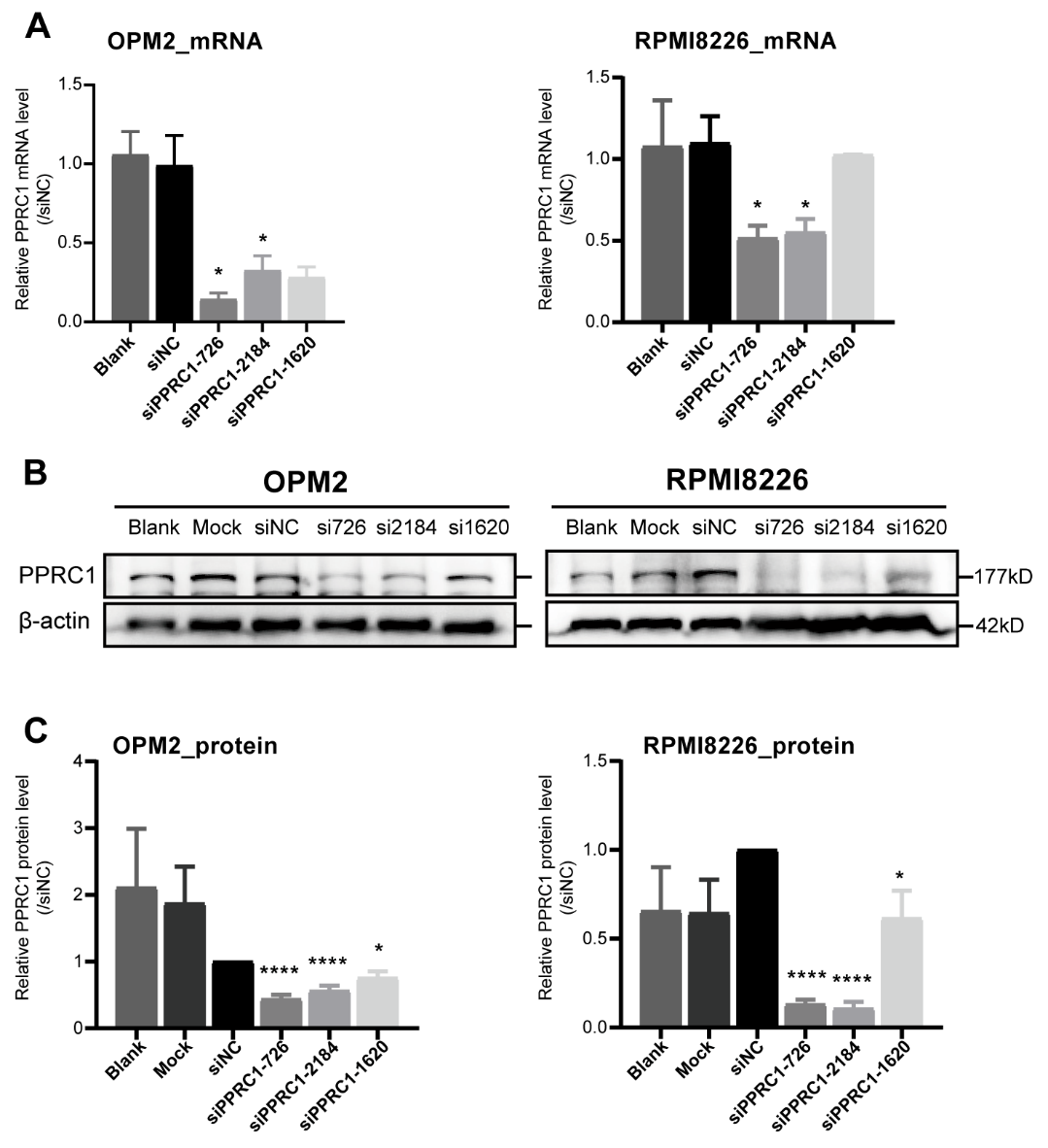


**Fig. S6** **PPRC1 knockdown efficiency in MM cells.** (A) PPRC1 mRNA level in control (Blank, Mock and siNC) and PPRC1 knockdown groups of OPM2 (left) and RPMI8226 (right) by RT-qPCR assay. (B-C) Western blot analysis and corresponding quantitative results of PPRC1 knockdown in OPM2 (left) and RPMI8226 (right) cells. PPRC1 was efficiently inhibited by siRNA transfection. β-actin is included as a loading control. Each experiment was repeated at least three times independently. Data are shown as the mean ± standard deviation. Blank, Mock and siNC are the wild type, transfection reagent and non-target vector control groups, respectively. **p*<0.05, ***p*<0.01, ****p*<0.001, *****p*<0.0001 compared to the siNC group.


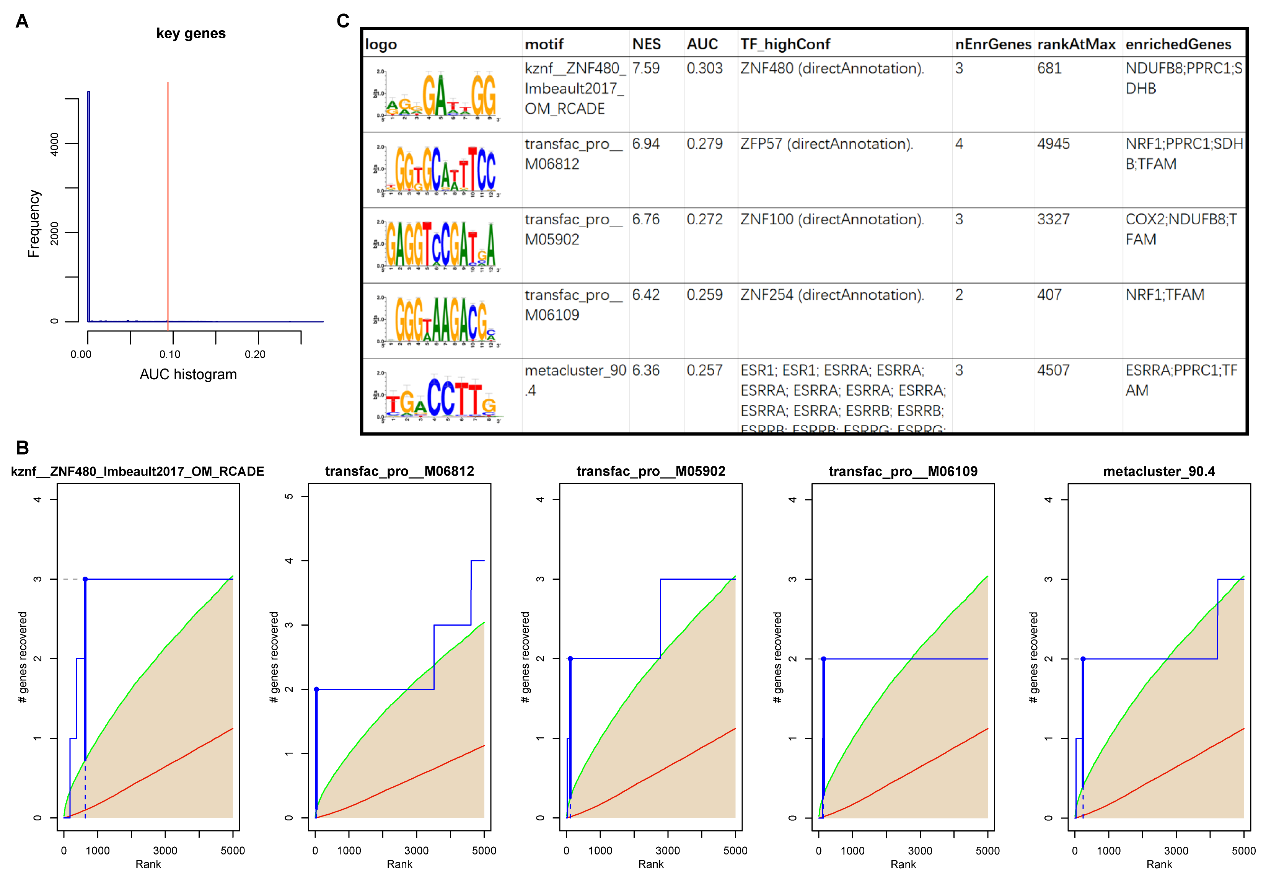


**Fig. S7** **Motif enrichment analysis for the tested OXPHOS-related genes.**

(A) AUC histogram. By calculating the AUC, the over-representation of each motif for key genes was assessed. Red line indicates the degree of significance, whereby motifs with AUC higher than the significance level are considered significant motifs. (B) The recovery curve for the top 5 most significant motifs. (C) Motif-TF annotation table showing the top 5 enriched motifs for key genes by “RcisTarget” R package. The motif “metacluster_90.4” is direct annotated to ESRRA transcription factor and enriched in 3 key genes including ESRRA.

**Reference**

1. Boiarsky, R., et al., *Single cell characterization of myeloma and its precursor conditions reveals transcriptional signatures of early tumorigenesis.* Nat Commun, 2022. **13**(1): p. 7040.

2. Yao, L., et al., *Single-Cell Discovery and Multiomic Characterization of Therapeutic Targets in Multiple Myeloma.* Cancer Res, 2023. **83**(8): p. 1214-1233.

3. Aibar, S., et al., *SCENIC: single-cell regulatory network inference and clustering.* Nat Methods, 2017. **14**(11): p. 1083-1086.

4. Cheng, Y., et al., *Multi-omics reveal immune microenvironment alterations in multiple myeloma and its precursor stages.* Blood Cancer J, 2024. **14**(1): p. 194.

5. Sklavenitis-Pistofidis, R., et al., *Immune biomarkers of response to immunotherapy in patients with high-risk smoldering myeloma.* Cancer Cell, 2022. **40**(11): p. 1358-1373.e8.

6. Tirier, S.M., et al., *Subclone-specific microenvironmental impact and drug response in refractory multiple myeloma revealed by single-cell transcriptomics.* Nat Commun, 2021. **12**(1): p. 6960.

7. Ru, B., et al., *TISIDB: an integrated repository portal for tumor-immune system interactions.* Bioinformatics, 2019. **35**(20): p. 4200-4202.

8. Danziger, S.A., et al., *Bone marrow microenvironments that contribute to patient outcomes in newly diagnosed multiple myeloma: A cohort study of patients in the Total Therapy clinical trials.* Plos Medicine, 2020. **17**(11): p. e1003323.
